# Acetylsalicylic acid modulates progression of endogenous thrombin potential in high-risk pregnancies

**DOI:** 10.1371/journal.pone.0347788

**Published:** 2026-04-21

**Authors:** Harald Haidl, Silvija Tokic, Eva-Christina Weiss, Siegfried Gallistl, Wolfgang Muntean, Axel Schlagenhauf

**Affiliations:** 1 Division of General Pediatrics, Department of Pediatrics and Adolescent Medicine, Medical University of Graz, Graz, Austria; 2 Department of Paediatrics and Adolescent Medicine, Medical University of Graz, Austria; 3 Research Unit Analytical Mass Spectrometry, Cell Biology and Biochemistry of Inborn Errors of Metabolism, Medical University of Graz, Austria; 4 Department of Obstetrics and Gynaecology, Medical University of Graz, Graz, Austria; Celal Bayar University: Manisa Celal Bayar Universitesi, TÜRKIYE

## Abstract

Pregnancy induces a hypercoagulable state peaking at delivery and reverting postpartum. This prospective cohort study evaluated the longitudinal progression of endogenous thrombin potential in 102 high-risk pregnant women in relation to the development of preeclampsia or gestational diabetes mellitus. Samples were collected from gestational weeks 8–15, with follow-ups every 2–12 weeks, and thrombin generation was assessed using Calibrated Automated Thrombography. Eleven women developed preeclampsia, and 19 developed gestational diabetes mellitus. Endogenous thrombin potential values were significantly elevated in patients who developed preeclampsia (2220 ± 357 nM*min, p < 0.001) or gestational diabetes mellitus (2298 ± 377 nM*min, p < 0.001) compared to the rest of the cohort (1995 ± 337 nM*min), with high levels evident from the first trimester—well before clinical symptoms. Notably, preeclampsia patients on acetylsalicylic acid therapy did not show further increases in endogenous thrombin potential, and acetylsalicylic acid intake in gestational diabetes mellitus patients effectively moderated endogenous thrombin potential progression. These findings suggest that higher early-pregnancy endogenous thrombin potential reflects an underlying hemostatic imbalance associated with the subsequent development of preeclampsia and gestational diabetes mellitus. Furthermore, acetylsalicylic acid appears to exert effects beyond its anti-inflammatory properties by moderating endogenous thrombin potential, providing new insights into the early pathophysiology and therapeutic modulation of high-risk pregnancies.

## Introduction

Pregnancy is associated with a progressive increase in hypercoagulability throughout gestation. Hypercoagulability peaks at parturition, and regresses within 4–6 weeks post partum [1]. This natural adaptation enhances a woman’s capacity to form blood clots more quickly and effectively, serving as a protective measure against potential bleeding [2].

While this prothrombotic shift is a physiological aspect of pregnancy, it is exacerbated in the presence of complications such as preeclampsia (PE) and gestational diabetes mellitus (GDM), amplifying the risk for arterial and venous thrombosis [3–6]. Numerous studies exploring the pathology of PE have concentrated mainly on markers indicative of angiogenic imbalance, a characteristic feature of PE that leads to platelet aggregation and triggers the hemostatic system [7]. Such markers, including placental growth factor (PLGF) and soluble fms-like tyrosine kinase 1 (sFLt1), have now been integrated into the screening protocols for PE [8]. The administration of low-dose acetylsalicylic acid (ASA) remains the gold standard for preventing excessive platelet activation and the development of PE [9,10].

Similarly, pregnant women diagnosed with gestational diabetes mellitus (GDM) exhibit endothelial dysfunction and face an elevated risk of thrombosis [5,11,12]. While factors such as obesity and hypertension may contribute to this risk, evidence from individuals with Type 1 diabetes—who are not universally affected by obesity or hypertension—reveals substantial endothelial disturbances [13–15]. This supports the notion that GDM itself may serve as an independent risk factor for endothelial dysfunction, thereby increasing thrombotic risk.

Collectively, endothelial dysfunction is implicated in the onset of both PE and GDM, potentially elucidating the heightened thrombosis risk observed in women with these conditions.

However, it is also crucial to consider how alterations in the balance of plasmatic hemostasis contribute to this increased risk. Throughout the natural course of pregnancy, there is a progressive increase in several clotting factors, including von Willebrand factor and factors VII, VIII, IX, X, and XII, while protein S levels decrease. Meanwhile, antithrombin and protein C levels remain unchanged [16–19]. Fibrinolysis is diminished as a result of reduced tissue plasminogen activator (t-PA) activity alongside increased levels of plasminogen activator inhibitor-1 (PAI-1) and the placenta-derived plasminogen activator inhibitor-2 (PAI-2) [20–22]. These adaptive mechanisms contribute to a progressive shift towards hypercoagulability that may become dysregulated during conditions such as PE or GDM [23].

However, conventional global coagulation tests, including prothrombin time (PT) and activated partial thromboplastin time (aPTT), fail to accurately reflect changes in levels of endogenous anticoagulant proteins throughout pregnancy, and thromboelastography often yields results within the normal range [24]. Those tests are not sensitive enough to detect subtle shifts towards hypercoagulability. In patients with PE, *in vivo* markers of thrombin generation, such as prothrombin fragments 1 + 2 (F1 + F2) and thrombin-antithrombin complexes (TAT), show elevated levels [25,26]. However, these markers cannot differentiate whether the observed increase in thrombin generation is due to influences from cellular components (e.g., endothelial perturbation and platelet activation) on coagulation or alterations within the plasmatic coagulation system itself.

The endogenous thrombin potential (ETP), as measured by calibrated automated thrombography, quantifies the total amount of thrombin that can be generated from an individual sample *in vitro*. This parameter is highly sensitive to pathological alterations within the coagulation system [27].

We hypothesized that the hemostatic abnormalities seen in women with pregnancy complications stem not solely from endothelial imbalance but from distinct alterations in plasmatic hemostasis. Therefore, we propose that women who develop PE and GDM show an atypical longitudinal pattern in their endogenous thrombin potential (ETP) throughout pregnancy. This pattern may provide insight into early plasmatic hemostatic alterations preceding the clinical manifestation of PE or GDM, thereby clarifying the role of plasmatic hypercoagulability in the early pathophysiology of these complications. To explore this hypothesis, we conducted a longitudinal study, using a retrospective diagnosis of PE and GDM to assess the changes in ETP over the course of pregnancy.

## Methods

### Patients and study design

Ethical approval for this study was granted by the ethics committee of the Medical University of Graz (25–568ex 12/13). All participants provided written informed consent prior to their inclusion. The study was conducted in accordance with the Declaration of Helsinki.

In this prospective cohort study, pregnant women attending routine check-ups at the high-risk pregnancy outpatient clinic of the Department of Gynecology and Obstetrics at the Medical University of Graz were invited to participate during a 12-month recruitment period from May 1st, 2014 to April 30th, 2015. Eligible participants were between 8 and 15 weeks of gestation at the time of recruitment. Following informed consent, an initial blood sample was collected, with subsequent samples taken at intervals ranging from 2 to 12 weeks during routine check-ups. Additional samples were collected at the time of PE diagnosis if it developed. Criteria for exclusion included the administration of heparin and the presence of severe pregnancy complications at the time of enrolment. Women with a positive first-trimester preeclampsia screening received a standardized dose of 100 mg ASA per day; adherence was documented for 67 patients. PE diagnosis adhered to the guidelines set by the International Society for the Study of Hypertension in Pregnancy (ISSHP) [28]. GDM diagnosis adhered to a consensus panel 2010 EK IV of the International Association of Diabetes and Pregnancy Study Group (IADPSG) based on results of the HAPO Study (Hyperglycemia and Adverse Pregnancy Outcome Study Cooperative Research Group 2009) [29].

### Laboratory analyses

Blood samples were collected via venipuncture into 4.5 ml Greiner VACUETTE® Sodium Citrate Coagulation Tubes, ensuring venostasis was not applied. Within one hour, the samples underwent a double centrifugation process (2600 × g, 10 minutes, 20°C) to obtain plasma, which was promptly frozen at −80°C for later analysis. Thrombin generation was assessed using Calibrated Automated Thrombography as reported previously with 5pM tissue factor (TF) as the initiator [14].

The following parameters were derived from the thrombin trace: Lagtime, ETP, Time to peak, Peak height, Start tail, and Velocity Index.

In addition, levels of D-dimer, prothrombin fragments 1 + 2 (F1 + F2), and thrombin-antithrombin complexes (TAT) were determined through commercial ELISA kits. The parameters for PE screening, soluble fms-like tyrosine kinase-1 (sFlt-1) and placental growth factor (PlGF), along with D-dimer, were measured using established routine methods.

### Statistics

Metric parameters are presented as mean ± standard deviation. Comparisons between groups were made using the mean of multiple data points per patient, analyzed with Student’s t-test or ANOVA, followed by Bonferroni post-hoc testing. To investigate the influence of gestational age, PE, GDM, and the intake of ASA on endogenous thrombin potential (ETP), a linear mixed-effects model was used. This model enabled the incorporation of both fixed effects (gestational age and the presence of PE/GDM or ASA intake) and random effects (variability among individuals) into our analysis. The analyses were conducted using the ‘statsmodels’ Python library. A p-value of less than 0.05 was deemed statistically significant.

## Results

Of the initial 121 pregnancies, 102 pregnancies were included in the final analysis. Exclusions were due to anticoagulation therapy, very early gestational age (week 6+) without follow-up measurements, or other severe complications. The dataset consisted of 42 visits in the first trimester, 155 in the second trimester, and 204 in the third trimester with an average of 3.9 ± 2.0 visits per patient. Eleven women developed PE and 19 women GDM during pregnancy. All but one preeclamptic patients developed symptoms after the second trimester. No symptomatic presentations occurred prior to 20 weeks of gestation. Blood of 10 women was drawn before clinical diagnosis of PE. Other risk factors in the cohort included a positive PE screening result (n = 70), PE or HELLP in previous pregnancies (n = 30), and obesity (n = 40). Some patients received antihypertensive therapy (n = 33), or ASA therapy (n = 67). Baseline characteristics of the cohorts are listed in Table 1.

**Table 1:** Demographic data of patient cohort.

	PE patients (n = 11)	GDM patients (n = 20)	Others (n = 71)
**Age at birth [years]**	35.1 (4.4)	32.9 (4.0)	‍34.6 (4.7)
**p-value**	0.693	0.120	‍-
**GA at delivery [weeks]**	36.9 (3.6)	39.0 (1.4)	‍38.8 (2.1)
**p-value**	0.115	0.684	‍-
**Birth weight [percentile]**	34.6 (26.1)	52.3 (31.1)	‍38.3 (26.5)
**p-value**	0.675	0.08	–
**ASA therapy [n (%)]**	9 (82%)	15 (75%)	43 (61%)

“Others” refers to the remaining study cohort consisting of high-risk pregnancies that did not develop PE or GDM. Data are represented as mean values with standard deviation. P-values indicate differences from Others determined using Student’s t-test

All women survived their pregnancies. The mean gestational age at delivery was 38.4 weeks, with 17 preterm births (<37 weeks), including 5 cases with preeclampsia and 3 births before 33 weeks. Birth weight distribution was analyzed using z-scores. Data were available for 93 neonates: 57 were below a z-score of 0, 23 below –1, and 6 below –2, while 9 were above a z-score of 1 and 1 above 2. Fourteen neonates required postnatal hospitalization: 10 due to prematurity, 1 for hyperbilirubinemia, 1 for bacterial infection, and 2 for adaptation difficulties (both with birth weights >90th percentile).

**Fig 1:**
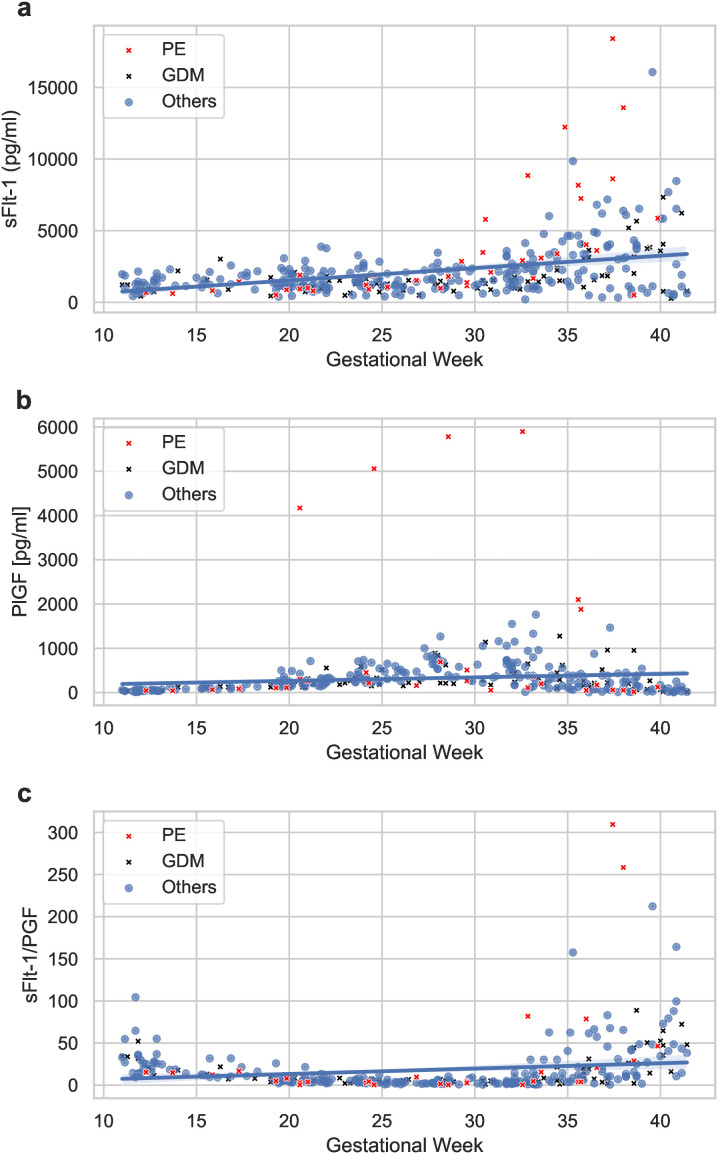
The progression of soluble fms-like tyrosine kinase-1 (sFlt-1) levels (a), placental growth factor (PlGF) levels (b), and the sFlt-1/PlGF ratio (c) throughout pregnancy within the cohort, inclusive of women who developed PE and GDM.

**Fig 2:**
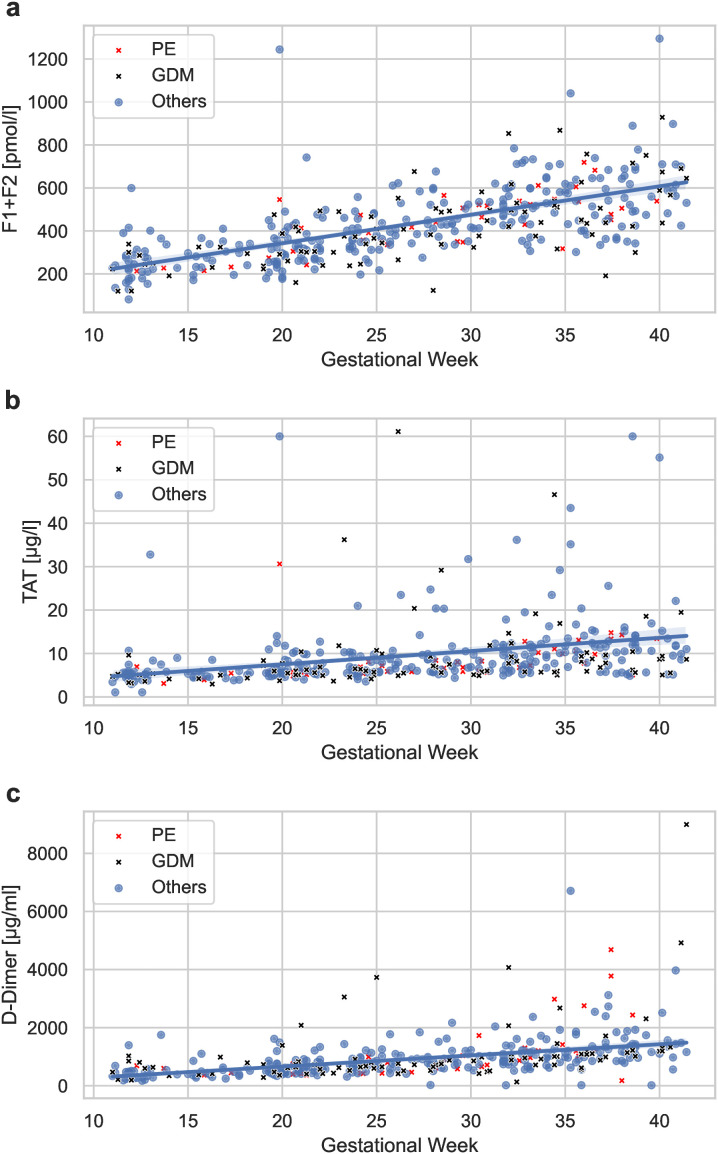
The progression of circulating levels of prothrombin fragments 1 and 2 (F1 + F2) (a), thrombin-antithrombin complexes (TAT) (b), and D-Dimer (c) throughout pregnancy within the cohort, inclusive of women who developed PE and GDM.

Longitudinal data showed a specific rise in sFLT-1, PlGF, and the sFLT-1/PlGF ratio in patients who developed PE indicating an angiogenic imbalance during the late third trimester (Fig 1a-1c). There was a continuous increase in coagulation activation markers across all women, with the most significant effects observed in F1 + F2. (Fig 2a-2c). However, there was no statistically significant difference in the progression of coagulation activation markers between women developing PE or GDM and those in other pregnancies.

**Fig 3:**
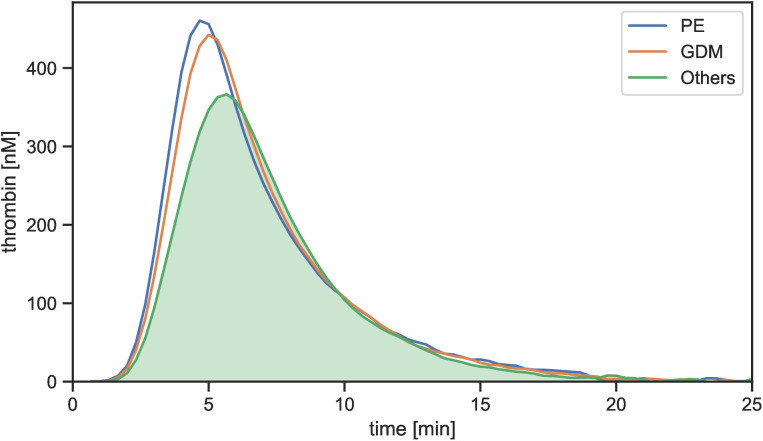
Representative thrombin traces showing profiles of women who developed PE, GDM, or the rest of the cohort (Others) and metric parameters derived from these traces.

Thrombin generation was significantly higher in women who developed PE and GDM compared to the rest of the cohort, particularly in terms of peak height and ETP (Fig 3, Table 2).

**Table 2:** Parameters derived from calibrated automated thrombin generation in patients with PE or GDM, compared to the rest of the cohort (Others).

	PE	p-value	GDM	p-value	Others
**Lagtime [min]**	2.58 (2.45-2.72)	0.936	2.65 (2.56-2.74)	0.160	2.58 (2.53-2.63)
ETP [nM*min]	2220 (2105-2336)	<0.001	2274 (2195-2353)	<0.001	2001 (1961-2041)
**Time to peak [min]**	5.19 (4.92-5.46)	<0.05	5.42 (5.24-5.60)	0.374	5.51 (5.41-5.60)
**Peak height [nM]**	434 (402-466)	<0.001	424 (405-442)	<0.001	372 (364-381)
**Start tail [min]**	22.7 ( 22.0-23.4)	0.661	23.0 (22.5-23.4)	0.798	22.9 (22.6-23.1)
**Vel. Index [nM/min]**	179 (157-202)	<0.001	164 (152-176)	<0.001	135 (129-140)

*Mean values with standard 95% confidence interval are presented. P-values indicate differences from Others using the mean of data points per patient and Student’s t-test*

**Fig 4:**
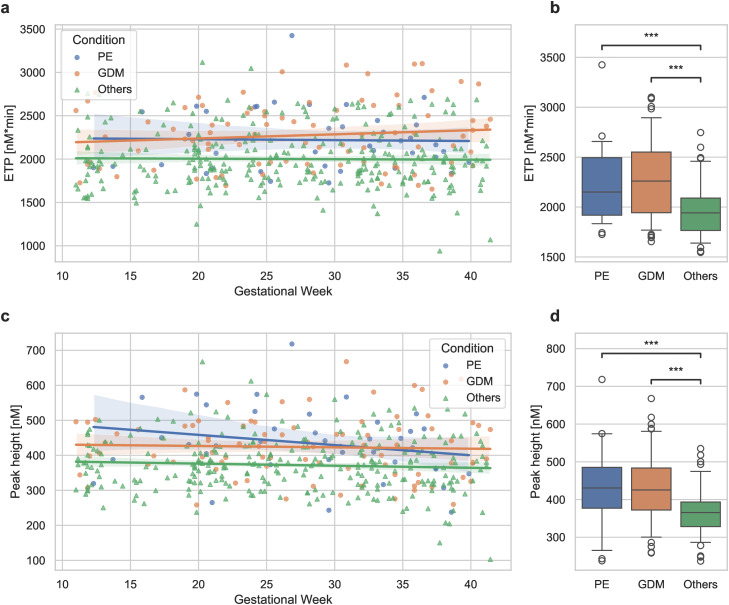
The progression of ETP (a) and Peak height (c) observed in thrombin generation traces during pregnancy among women who developed PE, GDM, or the rest of the cohort (Others). Shaded areas represent the 95% confidence interval for the regression estimate. The distribution of the means per patient is summarized in boxplots **(b, d)**. Statistical significance is denoted by ***p < 0.001.

Interestingly, these parameters were elevated in patients with PE and GDM compared to the rest of the cohort during the entire observation period and showed little change throughout the course of pregnancy (Fig 4a-4d). Linear mixed-effects models showed no impact of PE or GDM on the slope of the ETP progression (PE: p = 0.524; GDM: p = 0.868), but a significant impact on the intercept (PE: p < 0.001; GDM: p < 0.001), indicating that while these complications do not affect ETP progression, the absolute ETP values were consistently higher throughout the study period.

**Fig 5:**
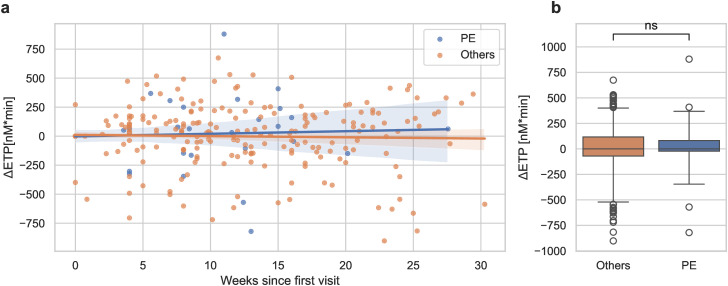
Change of ETP values (Δ ETP) since the first visit in women who developed PE vs. the rest of the cohort (Others) (a). Shaded areas represent the 95% confidence interval for the regression estimate. The distribution of the means per patient is summarized in boxplots **(b)**. ns: No statistical significant difference.

To further analyze the progression of ETP during pregnancy, the change in ETP (ΔETP) from the first visit was calculated at each subsequent visit for each participant. While women who developed PE had significantly higher absolute ETP values than others (Fig 4b), there was no further increase as indicated by ΔETP in individual patients during pregnancy, with progression similar to that of controls (Fig 5a, 5b). Notably, all but two PE patients had been identified as high-risk through positive PE screening results and consequently initiated on ASA therapy before the clinical onset of the disease.

**Fig 6:**
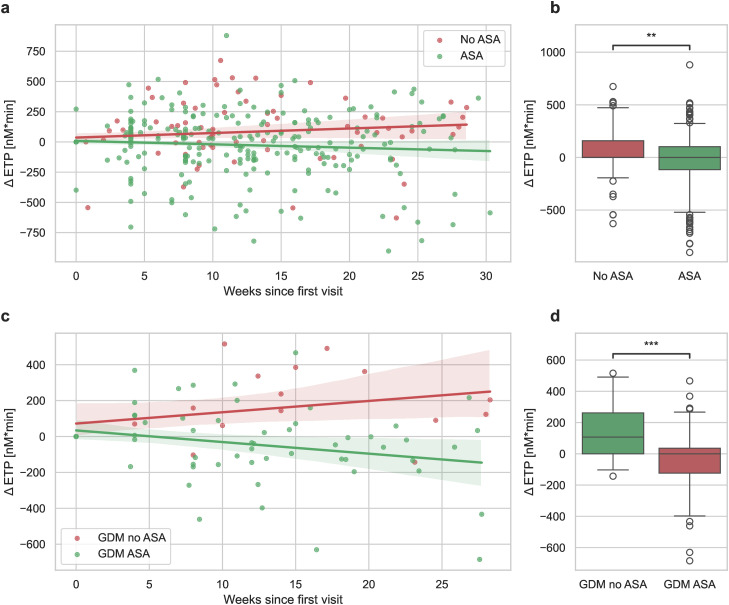
Changes in ETP values (Δ ETP) since the initial visit for the entire study cohort (a) and GDM patients (c), comparing patients taking ASA to those who are not. Shaded areas represent the 95% confidence interval for the regression estimate. The distribution of the means per patient is summarized in boxplots **(b, d)**. Statistical significance is denoted by **p < 0.01, ***p < 0.001.

When examining the entire cohort based on ASA intake (ASA: n = 67; no ASA: n = 35), there was a significant influence of ASA on the slope of ΔETP (linear mixed-effects model: p = 0.007)(Fig 6a, 6b). This finding indicates that ASA intake progressively lowers ETP values throughout pregnancy. When subgrouping women who developed GDM based on whether they received ASA therapy (n = 15) or not (n = 5), a significant difference in the progression of ΔETP was observed: ETP values increased in GDM patients not undergoing ASA therapy, while they decreased in those receiving ASA therapy (linear mixed-effects model: p = 0.005,)(Fig 6c, 6d).

The same effect of ASA therapy on ΔETP was observed in patients with obesity (ASA: n = 28, no ASA: n = 12; p = 0.022) (S1 Fig), and hypothyroidism (ASA: n = 22, no ASA: n = 10; p = 0.003) (S2 Fig).

## Discussion

We investigated the progression of thrombin generation in a cohort of high-risk pregnant women and observed that individuals who developed PE or GDM exhibited a higher ETP compared to women without pregnancy complications. Our findings are in agreement with another study showing that perturbations in thrombin generation identify women at risk of preeclampsia [30]. Parameters of thrombin generation have previously been shown to contribute to a composite score that successfully differentiates between early onset mild and severe PE and controls in a retrospective case-control study [31].

Although we observed no significant difference in the progression of ETP during pregnancy, the ETP in PE and GDM patients was consistently higher throughout the study period, starting in the first trimester, weeks before symptoms appeared and before the elevation of angiogenic imbalance markers. While it is well known that PE and GDM are associated with a prothrombotic state once they develop [5,6], our findings suggest that a pre-existing imbalance in the plasmatic hemostatic system may predispose individuals to these complications. This notion is further supported by the higher incidence of PE in thrombophilic patients [4].

Whereas ETP provides insight into hemostatic balance, F1 + F2 and TAT levels represent the hemostatic challenge by indicating thrombin generated *in vivo*. Interestingly, although F1 + F2 and TAT levels increased in all individuals during pregnancy, they were not significantly higher in those with PE and GDM compared to the rest of the cohort. This progressive rise in these markers may be a side effect of physiological changes caused by placental maturation and increasing blood flow. In contrast, ETP appears better suited to characterize the early plasmatic hemostatic imbalance associated with the subsequent development of these complications.

While the substantial inter-individual variability in absolute ETP values – consistent with previous reports [32]- limits its utility as a stand-alone predictive biomarker, the consistent longitudinal elevation observed in the PE and GDM groups suggests that an early prothrombotic shift in plasmatic hemostasis is a foundational component of the disease pathophysiology.

We observed a significant difference in the ETP progression among women who developed GDM and those with other pregnancy risk factors, based on their ASA intake. This effect was not observed in women who developed PE, as all but two individuals received ASA prophylaxis for most of their pregnancies. The effect of ASA on ETP is counterintuitive at first, as COX inhibition caused by ASA primarily affects platelets and the endothelium, while ETP is largely governed by clotting factors and inhibitors produced in the liver. Possibly, ASA alleviates endothelial disturbances associated with PE and GDM by inhibiting prostaglandin synthesis and Nuclear Factor–κB mobilization, thereby reducing the shedding of inflammatory cytokines such as IL-1, IL-6, and TNF-alpha in affected patients [33]. Consequently, it may lead to decreased hepatic secretion of acute-phase reactants, including prothrombotic factors such as factor VIII, and a reduction in antithrombin levels [34,35]. Additionally, endothelial cells can produce small amounts of factor V and factor II, which may be influenced by ASA’s anti-inflammatory effects on distressed endothelium [36]. Thus, ASA intake could affect not only primary hemostasis but also rebalance secondary hemostasis, reflected by a decrease in ETP.

A similar effect of ASA on ETP was observed in a study of patients with diabetes and acute coronary syndrome, showing a significant reduction in ETP after six months of taking 100 mg twice daily [37].

Our finding is particularly relevant because anticoagulation with heparin has been considered as an alternative or addition to ASA to delay the onset of PE [38,39]. Similarly, the use of heparin in treating GDM is currently under experimental investigation [40]. The therapeutic benefits of heparin, however, remain unclear; it is uncertain whether these benefits stem from its anticoagulant properties, its anti-inflammatory effects, or a combination of both [41,42]. Our data suggest that ASA, beyond its anti-inflammatory and platelet-inhibiting effects, can normalize plasmatic hemostasis without the added bleeding risk potentially associated with heparin. However, this observation needs to be confirmed in future studies, especially because there appears to be a significant subset of high-risk pregnancies that do not respond to low-dose ASA [43].

Our study has several limitations. Participants were recruited exclusively from a high-risk pregnancy outpatient clinic, which may limit the generalizability of our findings to low-risk populations typically managed in primary obstetric care settings. Consistent with our previous findings, women not taking ASA exhibited a modest increase in ETP over the course of pregnancy, a result that contrasts with the stable ETP levels reported by Hron et al. [32,44].

The high prevalence of ASA therapy within our PE cohort complicates the characterization of ‘baseline’ thrombin generation in these patients. While this reflects current clinical standards for high-risk pregnancies, it limits our ability to draw definitive conclusions regarding the unmodulated progression of ETP in women who develop preeclampsia without pharmacological intervention.

Additionally, other research suggests that thrombin generation may significantly escalate early in the first trimester, a factor potentially overlooked in previous publications and occurring before the observation period of this study [45].

## Conclusions

We conclude that an early prothrombotic shift in endogenous thrombin potential is a characteristic feature of pregnancies complicated by PE and GDM. While inter-individual variability restricts the use of ETP for routine risk prediction, our data reveal a significant modulatory effect of ASA on plasmatic hemostasis. This finding suggests a broader therapeutic mechanism for aspirin in high-risk pregnancies and underscores the need for further investigation into the interplay between anti-inflammatory therapy and secondary hemostasis.

## Supporting information

S1 Fig Changes in ETP values (Δ ETP) from the initial visit comparing obese patients taking ASA to those who are not. Shaded areas represent the 95% confidence interval for the regression estimate.
(DOCX)

S2 Fig Changes in ETP values (Δ ETP) from the initial visit comparing patients with hypothyroidism taking ASA to those who are not. Shaded areas represent the 95% confidence interval for the regression estimate.
(DOCX)

S1 File Maternal characteristics, angiogenic biomarkers, and thrombin generation metrics in the study cohort. Clinical and laboratory parameters of pregnant patients (n = 102), including demographic data (age, gestational age), screening status for preeclampsia (PE), and presence of comorbidities (Obesity, Hypothyroidism, GDM). The table presents plasma concentrations of soluble fms-like tyrosine kinase-1 (sFlt-1) and placental growth factor (PlGF), alongside the sFlt-1/PlGF ratio. Hemostatic profiles are detailed via calibrated automated thrombography (CAT) parameters, including endogenous thrombin potential (ETP), peak thrombin, lag time, and time to peak (ttPeak), as well as biochemical markers of fibrinolysis and coagulation activation (D-Dimer, TAT).
(PDF)
