# Supplementary material for: Acetylsalicylic acid modulates progression of endogenous thrombin potential in high-risk pregnancies
Source: PLoS One. 2026 Apr 21;21(4):e0347788. doi: 10.1371/journal.pone.0347788 (PMC13098978; doi:10.1371/journal.pone.0347788)
Supplement: S1 Fig — Shaded areas represent the 95% confidence interval for the regression estimate. (DOCX) [file pone.0347788.s001.docx]

**Supplementary Material**


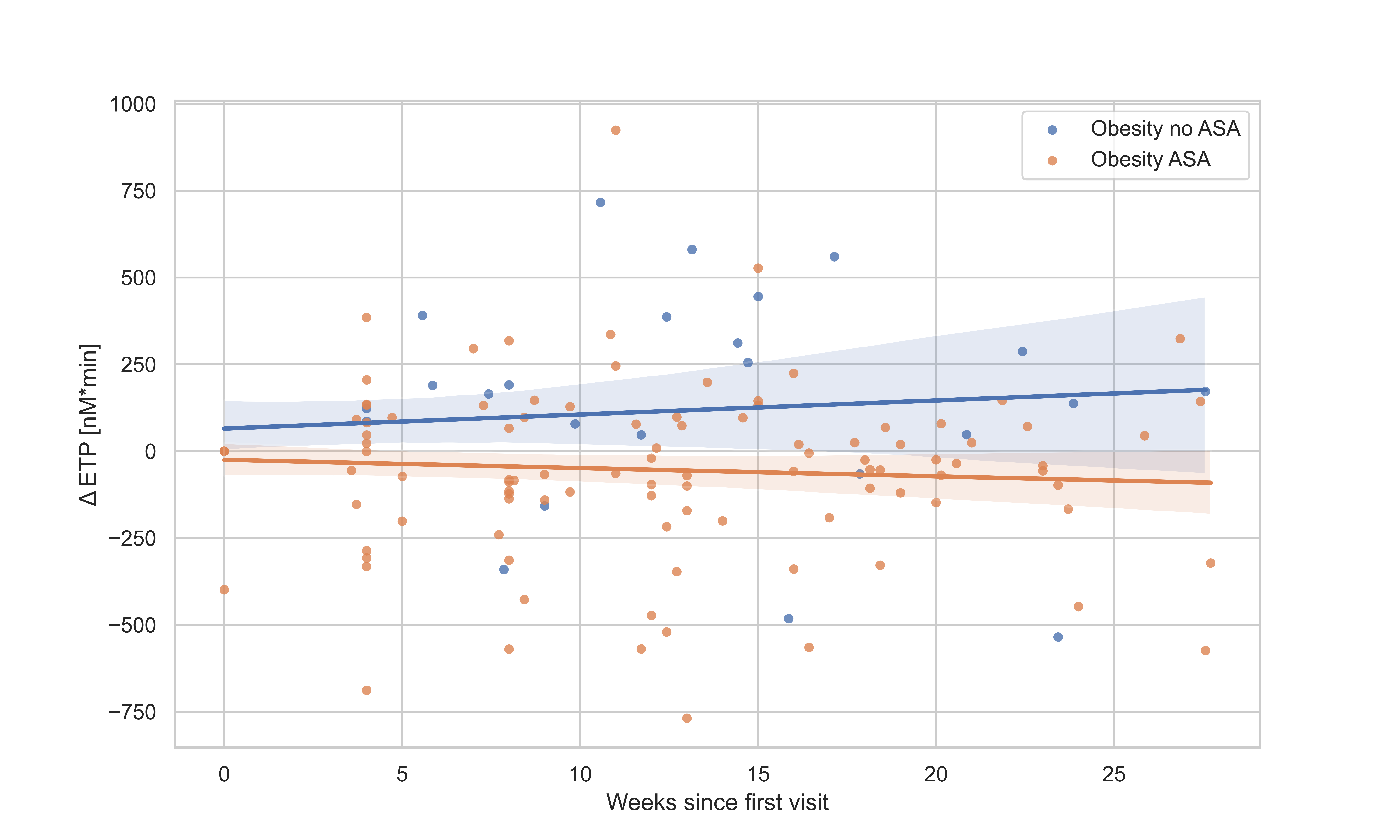


**S1 Fig:**

Changes in ETP values (Δ ETP) from the initial visit comparing obese patients taking ASA to those who are not. Shaded areas represent the 95% confidence interval for the regression estimate.
