# Supplementary material for: Acetylsalicylic acid modulates progression of endogenous thrombin potential in high-risk pregnancies
Source: PLoS One. 2026 Apr 21;21(4):e0347788. doi: 10.1371/journal.pone.0347788 (PMC13098978; doi:10.1371/journal.pone.0347788)
Supplement: S1 File — Clinical and laboratory parameters of pregnant patients (n = 102), including demographic data (age, gestational age), screening status for preeclampsia (PE), and presence of comorbidities (Obesity, Hypothyroidism, GDM). The table presents plasma concentrations of soluble fms-like tyrosine kinase-1 (sFlt-1) and placental growth factor (PlGF), alongside the sFlt-1/PlGF ratio. Hemostatic profiles are detailed via calibrated automated thrombography (CAT) parameters, including endogenous thrombin potential (ETP), peak thrombin, lag time, and time to peak (ttPeak), as well as biochemical markers of fibrinolysis and coagulation activation (D-Dimer, TAT). (PDF) [file pone.0347788.s003.pdf]

Patient_No	Age_at_due_date	PE	PE_Screening	Obesity	TASS	Hypothyroidism	Antihypertensive_Therapy	GDM	Child_GA_numeric	Child_GA	Gestational_Week	sFlt1_pg_ml	PGF	QsFlt-1/PlGf	Lagtime	ETP	Peak	ttPeak	VelIndex	StartTail	F1_F2	TAT	D-Dimer
69	33	0	1	1	1	1	0	0	39,28571429	39+2	27,85714286				2,67	2548,3265	446,48	5,57	154,84	24,73	24,73	24,73	24,73
71	26	0	1	0	1	0	1	0	39,57142857	39+4	22	845	223,3	3,784146888	2,33	1861,7978	275,35	6,11	73,04	24,89	307,6	10,22	900
72	39	0	1	0	1	1	1	0	40,28571429	40+2	15,28571429	1246	98,3	12,67548321	2,56	1620,1449	329,71	5,67	106,27	21,78	171,1	4,67	855
73	37	0	1	0	1	0	1	0	36,57142857	36+4	23,42857143	501			2,56	2200,0948	426,82	5	174,82	23,33	401,5	5,47	311
74	38	0	0	0	0	1	0	0	40,14285714	40+1	12,14285714	1367	52,9	25,84120983	2,33	1594,4495	316,15	5,33	105,38	22,44	287,2	5,78	500
75	32	0	1	0	1	0	1	1	39	39 + 0	20	969	153,8	6,300390117	3	2713,458	498,5	5,89	173,18	20,33	388,3	7,68	1390
76	39	1	1	1	1	0	1	0	36	36+0	15,85714286	816	66,7	12,23388306	2,89	2544,7677	565,74	5,11	256,79	20,89	214,5	3,92	365
77	41	0	1	0	1	0	0	0	39	39+0	19,85714286				2,33	1249,8473	237,21	5,67	71,16	20,89	1244,3	60	1490
79	37	0	1	0	1	0	0	0	38,71428571	38+5	17,71428571	2067	192,4	10,74324324	2,67	2574,3968	385,77	6,22	108,7	24,67	334,8	3,95	390
80	28	0	0	1	0	0	0	0	40,57142857	40+4	17,42857143	1872	87,3	21,44329897	2,67	2394,1054	449,41	5,22	176,79	24,67	195,4	3,83	475
82	36	0	0	0	0	0	1	1	38,28571429	38+2	14	2199	124,5	17,6626506	1,89	1796,9886	461,4	4	219,88	19,33	191,3	4,13	344
83	27	0	1	1	1	1	0	1	38,57142857	38+4	15,57142857	1560	119	13,1092437	2	2094,2463	473,11	4,22	214,2	21,22	325,2	4,21	376
85	43	0	1	0	1	0	1	0	37,42857143	37 + 3	20,14285714	1081			2,13	1462,5187	296,55	5,25	95,56	20,72	289,6	5,77	437
87	33	0	1	0	1	0	0	0		RG	11,85714286		16,6		2,45	2756,3081	459,2	5,12	171,75	25,4	223,5	5,94	1395
88	33	1	1	1	1	1	1	0	30,57142857	30 + 4	30,57142857	5793			3,56	2372,0398	408,48	6,56	145,12	25	464,1	8,23	654
89	32	0	1	1	1	0	1	1	37,85714286	37 + 6	11	1228	35,6	34,49438202	2,78	2560,2639	495,93	5,34	194,43	23,48	223,3	4,71	470
90	34	0	1	1	1	1	1	0	38	38+0	20	1345	126,3	10,64924782	2,67	2076,858	386,36	5,45	139,35	24,17	176,9	6,91	405
91	29	0	1	0	1	0	0	0	40,42857143	40 + 3	13,57142857	2306	125,5	18,37450199	3	2475,8565	435,19	6,01	144,67	21,94	365,1	7,47	1750
92	37	0	1	1	1	0	0	1	38,28571429	38 + 2	18,14285714			7,9	2,78	2344,7689	380,57	5,89	122,72	24,89	294,7	4,35	790
94	31	0	1	1	1	1	1	1	39,42857143	39 + 3	16,28571429	3024	138,9	21,8	3	2428,4798	374,19	6,33	113,72	26,67	230,2	2,95	415
98	32	0	1	0	1	1	0	0	37,28571429	37 + 2	16,85714286	2576	81	31,8	2,67	1743,3926	299,27	5,9	93,05	23,93	351,7	5,65	630
99	40	0	0	0	0	0	0	0	38,57142857	38+4	15,71428571	2124	83,2	31,8	2,44	1917,927	327,4	5,89	97,19	24,11	366,9	4,74	1100
100	41	0	1	0	1	1	0	0		RG	12,57142857	1017	64,9	15,7	2,44	1917,5016	412,56	5,01	161,43	23,06	179,1	3,87	285
103	28	0	0	0	0	0	1	0	37,85714286	37+6	19,71428571	1327			2,55	2131,2066	404,5	5,39	143,18	22,8	258,2	14,02	1194
104	26	0	1	1	1	0	1	1	40,14285714	40+1	32,42857143	912	368,6	2,474226804	4	2984,2589	457,1	7,01	152,13	22,03	530	8,22	130
105	30	0	1	1	1	1	1	1	40,28571429	40+2	12,71428571	740	64,1	11,54446178	2,28	1888,8223	406,41	5,06	146,91	21,53	244,3	3,58	599
106	30	0	1	0	1	1	0	0	41	41+0	11,71428571	873	13,5	64,66666667	2,45	1556,8574	288,85	5,67	89,7	23,26	285,5	4,42	365
107	37	0	0	0	0	1	0	0	32,28571429	32 + 2	11,57142857	435	29,9	14,54849498	1,67	1553,2667	378,32	4,11	155,55	19,56	390,3	5,6	185
108	28	0	0	1	0	0	0	0	38,85714286	38 + 6	13,85714286	1173	63,2	18,56012658	2,89	1944,8279	326,41	6,22	97,92	25,33	337	5,76	540
109	30	0	0	0	0	1	0	0	33,42857143	33 + 3	19,28571429			26	3,33	1785,9745	303,01	6,78	88,22	24	207	4,76	470
111	37	1	1	1	1	1	1	0	38	38+0	25,57142857				2,48	2656,8953	475,63	4,81	203,49	24,74	335,9	5,86	795
112	40	0	1	0	1	0	1	0	39	39 + 0	22	650	307	2,117263844	2,11	1612,0078	365,28	4,67	143,48	20,44	352,8	7,02	780
113	32	0	1	0	1	0	0	0	39,57142857	39+4	20			4,2	3	2111,508	435,11	5,44	178,79	22,44	185	5,45	570
114	38	0	1	1	1	0	1	1	39,42857143	39 + 3	21	1158	313,3	3,696137887	2,56	2392,4829	408,16	5,56	137,17	22,5	304,8	10,39	2080
115	40	0	0	1	0	0	0	0	40,28571429	40+2	11,71428571	639	67,5	9,466666667	2,22	1751,3574	379,89	4,67	155,87	21,92	276,1	5,07	474
116	40	0	1	0	1	0	1	0	40,57142857	40 + 4	15,71428571	1233	130,9	9,419404125	3	2551,077	476,78	5,56	187,21	24,84	230,3	5,79	494
117	29	0	1	0	1	0	1	0	38,42857143	38 + 3	28,28571429	906	701,7	1,291150064	2,33	1930,8686	370,12	5	138,79	22,44	372,7	5,2	1515
118	38	0	1	0	1	0	0	0	40	40+0	15,42857143	1280	108,2	11,82994455	2,33	1636,3156	284,97	5,78	82,97	22,89	281,3	4,53	277
119	38	0	0	0	0	0	0	0	40,71428571	40+5	12,14285714	663	34,1	19,44281525	2,67	1958,4495	365,54	5,33	137,08	24,33	244,7	3,47	255
120	31	0	0	1	0	1	0	0	41	41 + 0	11,85714286	1444	39,9	36,19047619	2,67	2143,4381	367,7	5,67	122,37	26,6	81,6	3,7	445
122	30	0	1	1	1	1	1	1	35,28571429	35 + 2	12	432			2,24	1964,9088	397,77	4,8	156,2	23,5	120,4	3,15	195
123	39	1	0	1	0	0	0	0	40,42857143	40 + 3	12,28571429	687	44,7	15,36912752	2,33	1901,3602	319,41	5,56	99,61	25	213	6,96	690
124	36	0	1	1	1	0	0	1	40,42857143	40 + 3	24,14285714	1634	322	5,074534161	2,67	2285,7483	495,04	5	212,16	22,17	245,5	6,39	643
125	31	0	1	0	1	0	0	0	40,85714286	40 + 6	16,28571429	1611			2,11	1812,9398	405,1	4,78	151,91	18,89	261,1	8,52	300
126	29	0	0	0	0	1	0	0		RG	12,71428571	1424	53,6	26,56716418	2,67	2746,4723	500,38	5,45	180,37	22,95	305,6	5,14	205
127	38	0	0	0	0	0	0	0	38,71428571	38 + 5	12,71428571	1584	56,3	28,13499112	2,78	1983,0123	339,17	6,12	101,47	23,95	277,4	5,21	470
127	29	0	1	0	1	0	0	0		RG	12	892	60,8	14,67105263	2,67	2254,7188	427,96	5,44	154,74	23,11	599,4	2,94	318
129	32	0	0	0	0	1	0	0	41,28571429	41+2	12,57142857	797	42	18,97619048	2,33	1866,3916	355,85	5,33	118,62	23,22	210,7	1,06	170
130	25	0	0	1	0	1	0	0	37,28571429	37 + 2	24,57142857			4,8	2,67	1698,1204	297,31	6	89,19	23,22	252,3	5,54	715
131	42	0	1	0	1	0	0	0		RG	19,71428571	3078	285,9	10,7660021	2,67	1898,4066	339,73	5,68	112,94	24,28	229,4	6,77	336
132	30	0	1	1	1	0	0	0	39,85714286	39 + 6	32,28571429	1152	432,4	2,664199815	3,33	2404,4282	428,93	6,11	155,09	24,33	486,9	9,41	792

133	38	0	1	1	1	0	1	1	38	38 + 0	21,71428571	959			2,1	1806,2664	381,36	4,78	145,11	20,84	304,6	5,58	722
134	40	0	0	1	0	0	1	0	39,71428571	39+5	11,85714286		15,7		3,33	1823,9881	298,37	6,78	86,87	25,11	258,4	10,63	485
135	35	0	0	0	0	0	0	0	36,42857143	36 + 3	36	3343	208,2	16,05667627	3	2285,1764	386,25	6,22	120,4	23,22	402,6	14,11	985
136	25	0	0	1	0	0	0	1	38,57142857	38 + 4	38,57142857	3180	71,3	44,6002805	2,33	1821,089	378,02	4,67	162,01	21,67	421,7	10,42	1295
137	40	0	1	1	1	1	0	0	38,85714286	38 + 6	20,28571429	1687			2,67	3115,8894	667,43	5	286,04	20,5	417,8	6,35	617
138	37	0	1	0	1	0	0	0	38,57142857	38 + 4	18,28571429	2167	133,8	16,19581465	2	1637,7496	342,2	5	114,07	20,78	340,4	5,59	515
139	41	0	1	0	1	0	0	0	37,71428571	37 + 5	15,28571429	1046	85,7	12,20536756	2,33	1555,8949	271,84	5,56	84,64	23,56	195,1	4,45	355
140	28	0	0	1	0	0	1	0	39,57142857	39 + 4	32,85714286	2624			2,33	2298,921	517,86	4,67	221,94	20,33	471,9	19,49	559
140	30	0	1	1	1	0	0	0	39,14285714	39 + 1	21,14285714				2,05	1900,0636	415,4	4,28	190,65	21,76	261,6	4	630
141	28	0	1	0	1	1	0	0	40	40+0	12,28571429	1178	46,4	25,38793103	2,33	1928,4002	417,7	5	156,64	20,78	312,9	4,34	269
142	30	1	1	0	1	1	0	0	40,71428571	40+5	21	1001	261,6	3,826452599	2,99	1890,2929	265,06	6,66	72,15	25,47	413,6	5,69	410
144	42	0	0	0	0	0	0	0	40,28571429	40 + 2	13		34,7		2,67	1659,1137	250,45	6,67	62,61	24,78	401,4	32,78	520
145	34	0	1	0	0	0	0	0	38	38 + 0	26,85714286	1436	475	3,023157895	1,89	2160,1016	414,69	4,67	162,96	22,33	342,3	6,85	905
147	38	0	0	0	0	0	0	0	37,57142857	37 + 4	11,85714286	2157	156,4	13,7915601	2,33	1545,6081	300,04	5,67	90,01	21,67	217,7	7,42	270
148	36	0	0	0	0	0	0	0	41	41 + 0	21,28571429	1605	210,6	7,621082621	2	1700,1243	328,28	5	109,43	22,89	741,8	9,81	565
149	31	0	1	1	1	0	1	1	38,85714286	38 + 6	11,85714286	1340	42,1	31,82897862	3,11	1938,1081	307,06	6,56	89,42	25,67	338,9	3,29	1025
152	41	1	1	1	1	1	0	0	37,42857143	37 + 3	21,28571429	801			2,36	2066,9543	427,01	4,8	175,27	22,5	242	5,15	355
153	36	0	0	0	0	0	1	0	39,14285714	39 + 1	29,28571429	2492			2,33	1743,1735	397,51	4,67	170,36	19,33	456,6	7,89	784
154	30	0	1	1	1	0	0	0	37,28571429	37 + 2	11,14285714	1013	37,4	27,0855615	2,89	2332,6346	389,89	6,12	121,19	23,25	135,1	1,06	449
155	38	0	0	0	0	0	0	0	41,14285714	41 + 1	12,85714286	2027	36,8	55,08152174	2,56	2090,133	372,99	5,33	134,49	23,78	209,9	3,73	340
156	36	0	0	0	0	0	0	1	41,14285714	41 + 1	13,14285714		39,2		3	2255,6444	435,83	5,67	163,43	22,78	250,5	5,37	635
157	40	0	1	0	1	0	0	0	38,14285714	38 + 1	11	1967	59,6	33,0033557	2,33	1701,5639	301,37	5,89	85,13	23,22	226,7	3,42	329
158	36	0	0	0	0	1	0	0	39,71428571	39+5	12	1187	39,7	29,89924433	2	1912,5388	474,5	4	237,25	21,22	181,6	5,28	510
159	37	1	1	0	1	0	0	0	40,28571429	40 + 2	30,42857143	3481			2,22	1859,1591	425,71	4,56	182,45	20,22	521,6	5,99	1728
161	34	0	0	1	0	1	1	1	39,28571429	39 + 2	22,14285714	1517			2,33	2403,9485	550,92	4,56	249,25	19,67	239,5	4,92	419
162	37	0	1	0	1	0	0	0	35,28571429	35 + 2	23,57142857	1532	231,2	6,626297578	2,25	2096,6155	349,6	5,48	108,6	24,63	327,1	6,94	725
163	42	0	1	0	1	0	0	0	36,14285714	36 + 1	11,85714286	1246	35,3	35,29745042	2,67	2129,1981	401,01	5,56	139,33	24,44	127,8	5,86	420
165	32	0	0	0	0	0	0	1	36,71428571	36 + 5	11,28571429	1221	36,1	33,82271468	2,33	1726,5053	344,08	5,22	119,64	21,67	119,8	5,22	215
167	35	0	1	1	1	1	0	1	38,57142857	38 + 4	20,71428571	1224	123,2	9,935064935	3	2111,6915	450,18	5,22	204,09	22,67	160	6	645
168	37	0	1	1	1	0	0	0	39,42857143	39+3	17				2	2290,1733	503,13	4,17	234,1	21,83	304,2	4,3	246
169	30	1	0	1	0	0	1	0	35,85714286	35 + 6	13,71428571	620	41,7	14,86810552	2,33	1916,2572	388,03	4,78	159,54	23,89	227,3	3,1	600
170	40	0	1	0	1	0	0	0	41,14285714	41 + 1	11,14285714	1866	34,1	54,72140762	2	1655,6246	373,07	4,67	139,9	20,89	240,1	4,66	685
171	34	0	1	0	1	0	1	0	33,14285714	33 + 1	11,71428571	1284	12,3	104,3902439	2,99	1981,7474	348,86	6,21	108,38	24,36	159,2	5,69	230
172	40	0	1	1	1	0	1	0	39,14285714	39 + 1	18,14285714	893			3	2649,4589	497,64	5,67	186,61	24	240,6	5,1	635
173	36	0	1	0	1	1	0	0	40,14285714	40 + 1	15,42857143	1567			2,44	1493,5756	322,59	5,56	104,19	20,44	232,4	5,27	343
174	38	1	1	0	1	0	0	0	39,71428571	39 + 5	20,57142857	945	301,5	3,134328358	2,33	2551,2508	544,31	5	204,12	19,33	420	4,82	395
175	30	0	0	0	0	1	0	0	35,85714286	35 + 6	29	1990			2,32	1729,3421	339,79	5,22	117,48	21,07	434,5	11,66	2168
176	33	0	0	0	0	0	0	0	39,42857143	39+3	12,85714286	1310	50,7	25,8382643	2,33	1945,083	404,21	5	151,58	22,67	272,2	4,71	540
177	28	1	1	1	1	0	1	0	30,85714286	30 + 6	17,28571429	1440	85,3	16,88159437	2,11	1935,4547	450,15	4,44	192,92	21,11	232,2	5,46	445
178	29	0	1	0	1	1	0	0	36	36 + 0	12,85714286	1718	46,7	36,78800857	2,22	1750,373	309,9	5,56	92,82	22,92	153,8	5,84	580
179	38	0	1	0	1	1	0	0	40,14285714	40+1	11,85714286				3,33	1573,1681	310,31	6,33	103,44	22,78	414,8	4,65	400
180	30	0	1	0	1	0	0	0	40,85714286	40 + 6	21,28571429	736	217,9	3,377696191	2,25	2176,6443	408,77	5,26	136,02	22,4	311,3	6,08	865
181	36	0	1	1	1	1	0	1	40,85714286	40 + 6	11,85714286	1027	19,7	52,1319797	3,33	2670,0581	494,72	6	185,52	24,22	300,5	9,62	850
182	37	0	1	1	1	1	0	1	40	40 + 0	12,42857143	953	46,2	20,62770563	3,11	2766,5309	502,32	5,67	197,19	24,67	286,2	5,09	805
183	35	0	0	0	0	0	0	0	33	33 + 0	24,42857143	1940	676,9	2,866006796	2	1556,2997	370,64	4,22	168,37	19,67	268	6,11	1218
184	31	0	0	0	0	0	1	0	40,42857143	40 + 3	21,85714286	2183	172,7	12,64041691	2	1767,9071	369,46	4,22	167,5	22,44	476,5	6,97	815
185	29	0	0	1	0	0	0	0	40,57142857	40 + 4	14,42857143	1138	81,4	13,98034398	3,33	2047,2607	312,15	6,67	93,64	27	228,3	8,99	390
186	27	0	1	1	1	0	0	0	40,28571429	40 + 2	15	1723			2,47	2114,3135	457,61	5,03	180,04	21,16	296,8	5,33	400
187	31	0	0	0	0	0	0	0	40,71428571	40+5	11,85714286				2,33	2485,9181	429,68	5,44	138,54	23,22	177,5	4,41	465
188	34	0	1	0	1	0	0	0	39	39+0	11,85714286	616	44,4	13,87387387	2,67	1979,8281	464,51	5,22	182,78	19,78	167,2	4,08	285
190	37	0	1	1	1	0	1	0	38,71428571	38 + 5	21,28571429	1226			2,33	2467,4443	524,39	4,83	210,38	21,33	385,6	6,95	674
69	33	0	1	1	1	1	0	0	39,28571429	39+2	31,85714286	1226	480,4	2,552039967	3	1843,9661	452,21	5,34	193,3	17,82	17,82	17,82	17,82

71	26	0	1	0	1	0	1	0	39,57142857	39+4	26	1034	259,3	3,987659082	2,44	1936,6174	280,31	6,22	74,32	25,22	406,2	7,38	1095
72	39	0	1	0	1	1	1	0	40,28571429	40+2	19,14285714	1368	213,4	6,41049672	2,33	2093,4838	426,66	4,89	167,82	22,22	179,4	5,51	495
73	37	0	1	0	1	0	1	0	36,57142857	36+4	25,71428571	590			2,33	2049,286	372,28	5,33	124,09	23,33	395,7	6,08	430
74	38	0	0	0	0	1	0	0	40,14285714	40+1	20,85714286	1807	232,5	7,772043011	1,98	1873,5922	383,24	4,66	143,43	22,25	316,6	5,87	515
75	32	0	1	0	1	0	1	1	39	39 + 0	32	1064	466,7	2,279837154	3	2678,7468	496,05	5,68	185,51	20,55	853,6	7,73	2065
76	39	1	1	1	1	0	1	0	36	36+0	19,85714286	878	109,5	8,01826484	2,89	2610,5073	574,74	5	273,82	21,89	546	30,62	405
77	41	0	1	0	1	0	0	0	39	39+0	25,85714286	1549	489,4	3,165100123	2	1767,8767	349,97	4,67	131,24	21,44	611,3	10,63	750
79	37	0	1	0	1	0	0	0	38,71428571	38+5	23,71428571	2354	706,8	3,330503679	3	2598,8462	377,7	6,33	118,09	25,89	325,5	5,54	930
80	28	0	0	1	0	0	0	0	40,57142857	40+4	25,28571429	1996	305,8	6,527141923	3	2022,0439	403,78	5,44	166,1	22,56	436,7	8,91	1015
82	36	0	0	0	0	0	1	1	38,28571429	38+2	22	1821	556,8	3,270474138	2	1693,9078	393,67	4,33	168,71	19,83	493,2	6,87	568
83	27	0	1	1	1	1	0	1	38,57142857	38+4	19,57142857	1485	252,3	5,885850178	2	2212,9859	485,33	4,22	219,84	21,22	475,6	5,96	474
85	43	0	1	0	1	0	1	0	37,42857143	37 + 3	25,14285714	1176			2,8	1785,1432	319,35	5,58	115,45	23,5	312,6	5,66	655
87	33	0	1	0	1	0	0	0		RG	22	3776	326,3	11,57217285	2,34	2036,6078	402,07	5,23	139,16	24,06	318,2	12,72	1335
89	32	0	1	1	1	0	1	1	37,85714286	37 + 6	19	1736	173,6	10	2	2593,9631	587,06	4	293,53	20,78	237	7,21	730
90	34	0	1	1	1	1	1	0	38	38+0	33	3442	113,5	30,32599119	2,33	1924,4417	375,46	5	140,8	21,78	335,5	5,86	675
91	29	0	1	0	1	0	0	0	40,42857143	40 + 3	19,57142857	2395	502,3	4,768066892	3,34	2527,9759	347,63	6,79	101,04	26,93	366,1	6,2	1615
92	37	0	1	1	1	0	0	1	38,28571429	38 + 2	27,85714286	1305	889,8	1,466621713	2,67	2188,3865	388,09	5,56	135,12	24,33	382,7	9,33	525
94	31	0	1	1	1	1	1	1	39,42857143	39 + 3	20,28571429	2106	250,2	8,417266187	2,67	2617,6894	395,2	6	118,56	26,33	260,1	5,48	620
98	32	0	1	0	1	1	0	0	37,28571429	37 + 2	20,85714286	2905	134,7	21,56644395	2,33	1895,4022	387,56	5	145,33	22,56	329,6	5,85	560
100	41	0	1	0	1	1	0	0		RG	20,57142857	993	426,7	2,32716194	2,33	2001,0408	402,3	5	150,86	23	426	10,14	680
102	35	1	1	0	1	0	1	0	35,71428571	35+5	20,57142857	1899	4170	0,455395683	2	1833,4008	404,64	4,33	173,42	20,44	305,2	5,76	755
103	28	0	0	0	0	0	1	0	37,85714286	37+6	21,71428571	1268	325,1	3,900338357	2,38	2146,8764	460,68	4,89	184,41	21,46	267,2	5,85	1214
104	26	0	1	1	1	0	1	1	40,14285714	40+1	36,42857143	1566	214,2	7,31092437	2,67	3100,8885	588,4	5	252,17	21	383,3	8,75	1105
105	30	0	1	1	1	1	1	1	40,28571429	40+2	16,71428571	900	126,1	7,137192704	2,28	1871,7819	398,35	4,95	149,1	20,98	324,6	5	985
107	37	0	0	0	0	1	0	0	32,28571429	32 + 2	20,71428571	1051	189	5,560846561	2	1671,5915	350,49	4,67	131,43	20,67	436,9	4,52	420
108	28	0	0	1	0	0	0	0	38,85714286	38 + 6	19,71428571	1124	195,3	5,755248336	2,67	2110,6366	366,74	5,67	122,25	24,67	323,4	5,07	515
111	37	1	1	1	1	1	1	0	38	38+0	29,57142857	1153	266,4	4,328078078	3,14	2354,0849	387,64	5,82	145,11	25,18	348	5,84	1035
112	40	0	1	0	1	0	1	0	39	39 + 0	26	486	518,3	0,93768088	2,33	1701,6974	388,2	4,67	166,37	20,67	379,1	6,51	810
113	32	0	1	0	1	0	0	0	39,57142857	39+4	24,14285714	906	365,7	2,477440525	2	2282,0083	461,6	4,67	173,1	23,33	197,1	5,81	985
114	38	0	1	1	1	0	1	1	39,42857143	39 + 3	25	1151	517,9	2,222436764	2,67	2399,0725	368,9	6,11	107,38	24,56	409,2	10,71	3730
115	40	0	0	1	0	0	0	0	40,28571429	40+2	21,57142857	899	152,3	5,902823375	2,33	1790,3757	425,2	4,33	212,6	20,44	354,6	5,83	435
116	40	0	1	0	1	0	1	0	40,57142857	40 + 4	19,71428571	1439	260,3	5,52823665	2,89	2059,3266	363,66	6	117,18	24,67	356,4	6,46	625
118	38	0	1	0	1	0	0	0	40	40+0	19,42857143	1136	257,8	4,40651668	2,11	1742,5952	325,64	5,33	101,3	22,11	315,9	5,86	585
119	38	0	0	0	0	0	0	0	40,71428571	40+5	20	977	166,3	5,874924835	2,32	2279,278	382,21	5,44	123,01	25,03	211,6	5,25	390
120	31	0	0	1	0	1	0	0	41	41 + 0	20,85714286	2382	259,9	9,165063486	2,22	1949,8522	353,61	5,22	117,87	24	260,8	6,94	640
121	32	0	1	0	1	0	0	0	38,14285714	38 + 1	13,14285714	1726	70,1	24,62196862	2,67	2428,2644	438,18	5,67	146,06	24	223,1	8,37	285
122	30	0	1	1	1	1	1	1	35,28571429	35 + 2	19	443	120,8	3,667218543	2,02	2231,7931	483,57	4,25	217,88	22,39	223,1	8,37	285
123	39	1	0	1	0	0	0	0	40,42857143	40 + 3	39,85714286	5871	126,3	46,48456057	2,44	1963,3353	347,37	5,33	120,44	23,67	538,7	13,44	
124	36	0	1	1	1	0	0	1	40,42857143	40 + 3	28,14285714	1483	215,8	6,8721038	2,33	2654,4579	559,83	4,56	254,38	22,11	504,6	6,81	715
125	31	0	1	0	1	0	0	0	40,85714286	40 + 6	20,28571429	1398			2,33	1776,2694	377,05	5,22	131,51	20,44	366	8,3	401
127	38	0	0	0	0	0	0	0	38,71428571	38 + 5	23,57142857	1430	237,6	6,018518519	2,1	1817,3555	347,39	5,1	115,57	22,47	316,1	10,29	515
127	29	0	1	0	1	0	0	0		RG	20	2320	251,5	9,224652087	2,67	2088,928	356,04	5,78	114,73	24	296,1	5,56	590
129	32	0	0	0	0	1	0	0	41,28571429	41+2	19,57142857	1172	130,2	9,001536098	2,56	1994,7259	373,95	5,33	134,98	23,33	257	4,98	375
130	25	0	0	1	0	1	0	0	37,28571429	37 + 2	28,57142857			10,4	2,33	1804,65	301,72	5,56	94,09	23,11	331	5,33	595
131	42	0	1	0	1	0	0	0		RG	23,71428571	3282	397,4	8,258681429	2,67	1932,6062	298,74	6,22	84,25	24,67	362,5	10,16	610
133	38	0	1	1	1	0	1	1	38	38 + 0	26,14285714	841	152,4	5,518372703	2,77	3006,9681	553,09	5,45	211,2	22,07	265,6	4,89	765
134	40	0	0	1	0	0	1	0	39,71428571	39+5	19,28571429	1371	203,6	6,733791749	3	1958,8445	286,39	6,78	76,02	26,22	450,1	10,95	750
137	40	0	1	1	1	1	0	0	38,85714286	38 + 6	23,85714286	1325			3,22	3046,2569	612,08	5,56	262,32	21,44	491,1	6,6	814
138	37	0	1	0	1	0	0	0	38,57142857	38 + 4	22,28571429	2171	298	7,285234899	2,22	1890,6792	312,4	5,67	90,78	22,81	498	5,7	573
139	41	0	1	0	1	0	0	0	37,71428571	37 + 5	19,28571429	891	173,4	5,138408304	2	1729,9845	327,36	5	109,12	22,67	254,9	5,66	860
140	28	0	0	1	0	0	1	0	39,57142857	39 + 4	35,85714286	3374			2,33	2495,1757	535,05	4,67	229,31	21,83	400,9	9,65	557
140	30	0	1	1	1	0	0	0	39,14285714	39 + 1	25,14285714	413	477,8	0,864378401	2,16	1950,8332	390,64	4,5	171,31	22,43	216,8	5,83	580

141	28	0	1	0	1	1	0	0	40	40+0	16,85714286	1276	123,1	10,36555646	2,33	1806,5526	365,04	5,22	126,87	21,22	410,3	5,49	541
142	30	1	1	0	1	1	0	0	40,71428571	40+5	29,57142857	1383	509,9	2,712296529	2,67	1725,7549	243,22	6,67	60,8	24,33	506,9	6,8	
144	42	0	0	0	0	0	0	0	40,28571429	40 + 2	21	942	261,3	3,605051665	2,89	2150,5229	381,33	5,78	132,51	23,22	282,6	7,12	410
145	34	0	1	0	0	0	0	0	38	38 + 0	32,14285714	2442	166,2	14,69314079	2	2605,0775	486,87	4,33	208,66	23,11	441,1	5,99	1370
148	36	0	0	0	0	0	0	0	41	41 + 0	32,14285714	1506	177,7	8,474957794	2,67	1943,5475	315,37	5,89	98,47	24,78	607,3	9,48	910
149	31	0	1	1	1	0	1	1	38,85714286	38 + 6	19,85714286	1465	128,4	11,40965732	2,67	1769,0273	258,26	6,44	69,47	24,78	291,6	3,73	364
152	41	1	1	1	1	1	0	0	37,42857143	37 + 3	25,28571429	1066			2,24	1743,4939	372,99	4,75	149,67	21,44	351,7	7,19	430
153	36	0	0	0	0	0	1	0	39,14285714	39 + 1	31,71428571	3584			2,67	1835,3854	348,81	5,67	116,27	21,33	474,9	9,65	865
154	30	0	1	1	1	0	0	0	37,28571429	37 + 2	16,14285714	632	70,3	8,990042674	2,78	2110,9491	367,34	5,89	118,42	23,81	338,9	4,56	390
155	38	0	0	0	0	0	0	0	41,14285714	41 + 1	21,71428571	3889	296,2	13,12964213	2,33	1865,2864	330,45	5,44	106,63	23,22	221,4	5,31	655
156	36	0	0	0	0	0	0	1	41,14285714	41 + 1	23,28571429	657	266,8	2,462518741	3	2771,2841	483,54	5,67	181,33	23,56	375,5	36,21	3055
157	40	0	1	0	1	0	0	0	38,14285714	38 + 1	16				3	1876,7284	261,94	7	65,49	26,11	274,6	4,74	440
159	37	1	1	0	1	0	0	0	40,28571429	40 + 2	34,42857143	3392			2,67	1895,0987	380,94	5,44	137,7	20,78	548	11,04	2980
161	34	0	0	1	0	1	1	1	39,28571429	39 + 2	26,14285714	1160			2,67	2473,8081	458,65	5,56	159,4	23,11	552,4	61,11	414
162	37	0	1	0	1	0	0	0	35,28571429	35 + 2	28,57142857	2241	394,5	5,680608365	2,25	2240,9	389,79	5,26	129,7	23,74	531,2	20,32	935
163	42	0	1	0	1	0	0	0	36,14285714	36 + 1	19,71428571	2203	358,5	6,145048815	2,67	2166,4866	370,41	5,78	119,39	24,11	248,6	6,18	750
165	32	0	0	0	0	0	0	1	36,71428571	36 + 5	21,28571429	1103	202,6	5,444225074	2	1787,7743	360,08	4,67	135,03	22,44	301,1	6,24	400
167	35	0	1	1	1	1	0	1	38,57142857	38 + 4	24,42857143	1124	150,8	7,453580902	2,67	1943,8997	425,88	5,01	182,23	22,14	339,5	5,99	675
169	30	1	0	1	0	0	1	0	35,85714286	35 + 6	19,28571429	514	104,2	4,932821497	2,32	2284,5145	430,61	4,66	184,19	24,25	275	5,86	565
170	40	0	1	0	1	0	0	0	41,14285714	41 + 1	19,71428571	1596	158,8	10,05037783	3,33	1871,2766	321,63	6,67	96,49	23,56	242,8	12,41	765
172	40	0	1	1	1	0	1	0	39,14285714	39 + 1	22,14285714	995	338	2,943786982	3	2679,9085	509,31	5,56	200,31	23,89	461,8	7,81	570
173	36	0	1	0	1	1	0	0	40,14285714	40 + 1	17,71428571	1820			2,33	1625,2768	335,79	5,44	108,5	20,78	297,4	5,99	341
174	38	1	1	0	1	0	0	0	39,71428571	39 + 5	24,14285714	1217	453	2,686534216	2,67	2601,9983	525,32	5	225,14	20,83	474	6,9	435
176	33	0	0	0	0	0	0	0	39,42857143	39+3	21,85714286	1325	257,9	5,137650252	2,65	1941,7671	364,12	5,32	136,28	23,36	321,2	5,16	360
178	29	0	1	0	1	1	0	0	36	36 + 0	20,71428571	1196	251,2	4,761146497	2,11	1775,2515	371,26	5	128,75	21,33	328,6	10,49	886
179	38	0	1	0	1	1	0	0	40,14285714	40+1	21,14285714	2204	711	3,099859353	3	1876,4336	354,01	6	118	23,11	318	9,88	915
180	30	0	1	0	1	0	0	0	40,85714286	40 + 6	25,28571429	1071	653,2	1,639620331	2,25	2217,0039	401,15	5,15	140,78	22,62	362,8	7,89	1255
181	36	0	1	1	1	1	0	1	40,85714286	40 + 6	24,85714286	1549	242,2	6,395540875	3	2548,0018	459,46	5,67	172,3	24,78	361,8	5,04	
182	37	0	1	1	1	1	0	1	40	40 + 0	20,85714286	736	113,8	6,467486819	2,89	2305,4422	381,13	5,89	127,04	24,56	399,8	5,1	830
183	35	0	0	0	0	0	0	0	33	33 + 0	27,42857143	1590	816,3	1,947813304	2	1729,7044	390,07	4,33	167,17	19,78	275,6	9,68	914
184	31	0	0	0	0	0	1	0	40,42857143	40 + 3	31,85714286	2103	643,6	3,267557489	2,33	1872,9861	409,12	4,33	204,56	21	598,4	7,26	1385
186	27	0	1	1	1	0	0	0	40,28571429	40 + 2	20	1982			2,69	2021,628	380,97	5,69	126,77	22,94	385,1	5,66	494
187	31	0	0	0	0	0	0	0	40,71428571	40+5	21,71428571	1857	139,7	13,29277022	2,67	2458,5564	340,87	6,45	90,28	25,26	273,9	5,44	515
188	34	0	1	0	1	0	0	0	39	39+0	19,42857143	392	174,6	2,24513173	2,56	2385,3352	521,34	5	214,44	20,44	421,9	4,74	422
190	37	0	1	1	1	0	1	0	38,71428571	38 + 5	25	1118			2,22	2544,1725	573,83	4,33	273,58	20,33	381,4	8,47	858
69	33	0	1	1	1	1	0	0	39,28571429	39+2	35,85714286	2249	367,9	6,113074205	3	1946,4057	439,48	5,46	180,22	20,38	20,38	20,38	20,38
71	26	0	1	0	1	0	1	0	39,57142857	39+4	31	1550	337,7	4,589872668	2,67	1974,3019	263,79	6,78	64,31	25,67	534,2	6	1030
72	39	0	1	0	1	1	1	0	40,28571429	40+2	22	1119	243,9	4,587945879	2,34	1727,2578	320,47	5,34	106,65	22,26	259,7	7,36	660
73	37	0	1	0	1	0	1	0	36,57142857	36+4	26,57142857	837			2,33	2067,9602	379,29	5,33	126,43	23,17	453	6,39	410
75	32	0	1	0	1	0	1	1	39	39 + 0	34,71428571	1486	618,1	2,404141725	3,33	2618,8401	473,95	5,89	186,35	22,56	868,1	16,92	2675
76	39	1	1	1	1	0	1	0	36	36+0	24,28571429	918	217,1	4,228466145	2,67	2608,199	574,3	5	246,13	21,33	357,5	5,36	415
79	37	0	1	0	1	0	0	0	38,71428571	38+5	27,71428571	2455	838	2,929594272	3	2601,2458	344,16	6,67	93,86	27,11	431,6	8,67	990
80	28	0	0	1	0	0	0	0	40,57142857	40+4	33,28571429	2493	215,9	11,54701251	2,67	1848,2331	382,45	5,22	150,73	21,67	505,7	10,39	1185
82	36	0	0	0	0	0	1	1	38,28571429	38+2	28	1237	841,9	1,469295641	1,89	1941,0772	437,67	4	208,26	21	123,2	7,14	654
83	27	0	1	1	1	1	0	1	38,57142857	38+4	30,57142857	1302	1140	1,142105263	2	2166,2698	460,05	4,56	180,85	21,22	582	5,79	986
85	43	0	1	0	1	0	1	0	37,42857143	37 + 3	29,14285714	1510			2,08	1411,0128	286,18	4,92	101,41	20,44	399	6,06	710
87	33	0	1	0	1	0	0	0		RG	34,71428571	4260	555,2	7,672910663	2,56	1853,8301	424,94	5,01	174,15	21,83	711	29,2	1925
89	32	0	1	1	1	0	1	1	37,85714286	37 + 6	23,85714286	1587	589	2,694397284	2,89	2582,2769	497,9	5,22	213,39	22,22	373,3	6,47	915
90	34	0	1	1	1	1	1	0	38	38+0	36,42857143	4909	74	66,33783784	3	2005,2485	325,89	6,22	101,72	24,67	514,2	15,36	850
91	29	0	1	0	1	0	0	0	40,42857143	40 + 3	23,28571429	2061	390,8	5,273797339	2,67	2504,6941	482,22	5	206,66	21,33	460,8	10,28	1435
92	37	0	1	1	1	0	0	1	38,28571429	38 + 2	35,85714286	4076	265,9	15,32907108	3,45	2298,3857	312,32	7,12	85,04	26,93	445,7	8,17	1090
94	31	0	1	1	1	1	1	1	39,42857143	39 + 3	23,57142857	1280	321,9	3,976390183	3,33	2530,6155	327,66	7,01	89,23	29,93	238	4,56	528

98	32	0	1	0	1	1	0	0	37,28571429	37 + 2	24,85714286	2842	227,1	12,51431088	2,56	1627,3118	297,36	5,56	99,12	21,78	478,9	9,78	820
102	35	1	1	0	1	0	1	0	35,71428571	35+5	24,57142857	1906	5057	0,376903302	1,89	1855,2104	439,88	4	209,39	19,44	391,1	8,1	993
103	28	0	0	0	0	0	1	0	37,85714286	37+6	25,71428571	1254	330	3,8	2,71	2037,826	430,03	5,22	172,45	22,13	335,2	8,68	1772
105	30	0	1	1	1	1	1	1	40,28571429	40+2	20,71428571	1085	171,4	6,330221704	2,39	2174,6215	442,48	5,06	165,61	20,87	418,7	5,09	735
108	28	0	0	1	0	0	0	0	38,85714286	38 + 6	31,71428571	829	726,6	1,140930361	2,67	1807,7554	316,85	5,89	98,79	23,11	515,8	6,13	820
111	37	1	1	1	1	1	1	0	38	38+0	33,57142857	3093	199,2	15,52710843	3,03	2310,9145	316,53	6,59	89,31	27,19	611,7	10,2	1205
112	40	0	1	0	1	0	1	0	39	39 + 0	39	968	541,5	1,787626962	2,33	1379,5811	326,37	4,33	163,19	19,44			
114	38	0	1	1	1	0	1	1	39,42857143	39 + 3	32	1516	477,2	3,176865046	2,65	2284,2168	312,29	6,55	80,41	25,81	420,3	14,65	4070
115	40	0	0	1	0	0	0	0	40,28571429	40+2	35,57142857	1552	126,1	12,30769231	2,67	1793,0243	380,54	5	163,09	21,56	301,8	16,67	805
116	40	0	1	0	1	0	1	0	40,57142857	40 + 4	24	1741	593	2,935919056	2,89	2218,7476	437,57	5,33	179,96	23,78	394,9	5,86	660
118	38	0	1	0	1	0	0	0	40	40+0	25,71428571	1134	598,5	1,894736842	2,33	1856,416	355,78	5,33	118,59	21,33	350,4	9,08	585
119	38	0	0	0	0	0	0	0	40,71428571	40+5	32	1085	569,5	1,905179982	2,67	2086,6468	342,55	5,89	106,65	24,11	424,9	5,56	
120	31	0	0	1	0	1	0	0	41	41 + 0	32,71428571	2312	583,2	3,964334705	2,67	2107,3603	383,21	5,44	138,49	23,44	354	6,27	1220
121	32	0	1	0	1	0	0	0	38,14285714	38 + 1	20,14285714	2006	489,1	4,101410754	2,34	1925,4587	321,51	5,79	93,48	23,7	489,9	11,78	623
122	30	0	1	1	1	1	1	1	35,28571429	35 + 2	23	487	219,2	2,221715328	2,35	2166,1027	432,96	4,58	195,66	23,72	489,9	11,78	623
124	36	0	1	1	1	0	0	1	40,42857143	40 + 3	32,14285714	1746	442,4	3,946654611	2,44	2130,7875	428,42	5	168,34	22,22	496,9	9,25	885
125	31	0	1	0	1	0	0	0	40,85714286	40 + 6	23,71428571	1568			2	1873,3262	395,92	4,89	137,28	20,11	411,1	6,52	455
127	29	0	1	0	1	0	0	0		RG	24	2573	285,5	9,012259194	3	2411,1876	437,98	5,56	171,97	24,03	456,6	4,71	720
129	32	0	0	0	0	1	0	0	41,28571429	41+2	24,42857143	1229	215,7	5,697728326	2,67	2280,7397	405	5,45	146,22	24,14	350,7	3,55	484
130	25	0	0	1	0	1	0	0	37,28571429	37 + 2	35,14285714	4645	74,4	62,4327957	2,67	2372,1122	408,57	5,33	153,21	24,11	431,6	13,48	965
131	42	0	1	0	1	0	0	0		RG	28	3469	535,3	6,480478237	2,78	1851,0672	293,51	6,22	85,43	24,44	410,9	8,65	840
133	38	0	1	1	1	0	1	1	38	38 + 0	30,85714286	888	171,7	5,171811299	2,21	3084,5812	667,74	4,56	284,99	18,5	447,9	5,98	478
134	40	0	0	1	0	0	1	0	39,71428571	39+5	26,28571429	2089	440,1	4,746648489	3	2077,5088	331,87	6,23	103,18	24,26	529,7	23,48	1036
138	37	0	1	0	1	0	0	0	38,57142857	38 + 4	32,28571429	2188	683,1	3,203044942	2,33	1884,9082	326,55	5,44	105,35	21,44	784,3	8,33	516
139	41	0	1	0	1	0	0	0	37,71428571	37 + 5	23,85714286	1122	332,6	3,373421527	2,78	1764,7269	286,67	6	89,1	24,25	303,8	5,33	715
140	30	0	1	1	1	0	0	0	39,14285714	39 + 1	29,14285714	499	582,7	0,856358332	3	2178,0728	414,61	5,33	177,69	24,22	336,7	4,38	930
141	28	0	1	0	1	1	0	0	40	40+0	20,14285714	1915	207,7	9,220028888	2,33	1976,1187	406,69	5	152,51	21,17	397,4	8,55	535
144	42	0	0	0	0	0	0	0	40,28571429	40 + 2	35	1448	395,4	3,662114315	2,78	1736,4415	330,75	5,78	110,25	21,11	580,2	11,71	990
145	34	0	1	0	0	0	0	0	38	38 + 0	37,14285714	7185	86,5	83,06358382	2,33	2632,762	407,43	5,33	135,81	26,67	362,9	5,75	1335
149	31	0	1	1	1	0	1	1	38,85714286	38 + 6	22,71428571	1527	181	8,436464088	3	2230,4813	320,77	6,56	90,45	25,59	300,5	3,64	434
152	41	1	1	1	1	1	0	0	37,42857143	37 + 3	29,28571429	2866			2,58	1919,9435	441,47	4,75	205,04	21,11	350,8	8,01	580
153	36	0	0	0	0	0	1	0	39,14285714	39 + 1	32,71428571	4135			2,33	1841,8003	363,38	5,33	121,13	20,67	453	7,07	1094
154	30	0	1	1	1	0	0	0	37,28571429	37 + 2	20,14285714	455	155,3	2,929813265	2,67	2229,7087	379,24	5,78	122,43	24,56	281,9	4,59	436
155	38	0	0	0	0	0	0	0	41,14285714	41 + 1	36,85714286	6197	383,3	16,16749283	3,22	1741,0006	247,52	7	65,68	24,22	334,5	11,31	935
156	36	0	0	0	0	0	0	1	41,14285714	41 + 1	41,14285714	6230	86,2	72,2737819	2,67	2379,3816	389,93	5,67	129,98	24,67	690,2	19,49	4920
157	40	0	1	0	1	0	0	0	38,14285714	38 + 1	24	2400	737,1	3,256003256	3	1989,2876	285,41	6,78	75,59	25,7	414	4,09	429
159	37	1	1	0	1	0	0	0	40,28571429	40 + 2	37,42857143	8624			2,56	2165,4234	363,3	5,89	110,51	23,56	452,1	13,35	3777
161	34	0	0	1	0	1	1	1	39,28571429	39 + 2	30,14285714	979			2,78	2562,5277	460,13	5,67	159,87	23	323,6	5,11	855
162	37	0	1	0	1	0	0	0	35,28571429	35 + 2	29,85714286	2368	260,1	9,104190696	2,36	2462,3363	399,02	5,59	124,15	24,18	352	31,74	1085
163	42	0	1	0	1	0	0	0	36,14285714	36 + 1	23,71428571	1475	469,9	3,138965737	2,33	2284,5962	427,53	5	160,32	24,56	355	9,36	625
165	32	0	0	0	0	0	0	1	36,71428571	36 + 5	25,28571429	1294	294,4	4,395380435	2,33	1963,4039	393,43	5	147,54	21,67	344,4	9,89	650
167	35	0	1	1	1	1	0	1	38,57142857	38 + 4	28,42857143	1067	212	5,033018868	3	1840,1193	424,82	5	212,41	21,56	337,8	5,61	800
172	40	0	1	1	1	0	1	0	39,14285714	39 + 1	26,14285714	1102	462,5	2,382702703	2,44	2534,7681	498,13	5	195,91	23	622,5	7,96	532
173	36	0	1	0	1	1	0	0	40,14285714	40 + 1	20,85714286	1581			2,33	1610,0922	316,86	5,67	96,38	21	332,9	7,88	517
174	38	1	1	0	1	0	0	0	39,71428571	39 + 5	28,14285714	984	692,8	1,420323326	2,67	2629,9479	517,7	5,33	194,14	20,67	439,9	8,4	615
179	38	0	1	0	1	1	0	0	40,14285714	40+1	33,14285714	2125	1330	1,597744361	2,67	1662,6024	303,23	5,67	101,08	22,56	599,3	11,38	1515
180	30	0	1	0	1	0	0	0	40,85714286	40 + 6	33,28571429	1134	1762	0,643586833	2,48	2220,0231	427,91	5,15	160,19	21,4	733,5	11,5	2370
182	37	0	1	1	1	1	0	1	40	40 + 0	24,85714286	726	181,9	3,991203958	2,89	2499,1818	440,02	5,56	165,01	25,33	365,8	5,73	600
184	31	0	0	0	0	0	1	0	40,42857143	40 + 3	35,57142857	1999	389,7	5,129586862	2,56	1779,0143	364,09	5	149,82	21,67	638,6	8,81	1860
186	27	0	1	1	1	0	0	0	40,28571429	40 + 2	24	2737			2,58	1937,5076	374,38	5,58	124,58	22,28	557,7	20,98	765
187	31	0	0	0	0	0	0	0	40,71428571	40+5	34,71428571	2262	157,3	14,38016529	2,67	2598,8501	493,15	5,33	184,93	21,5	580,3	10,07	810
188	34	0	1	0	1	0	0	0	39	39+0	30,42857143	609	1053	0,578347578	2,67	2183,6091	468,42	5	200,75	20,89	534,6	6,57	1024

190	37	0	1	1	1	0	1	0	38,71428571	38 + 5	30	1162			2,5	2579,317	559,91	4,5	279,96	21,5	416,7	6,89	1227
69	33	0	1	1	1	1	0	0	39,28571429	39+2	39,57142857	1122	23,2	48,36206897	2,34	1931,8539	518,21	4,34	258,43	16,93	16,93	16,93	16,93
71	26	0	1	0	1	0	1	0	39,57142857	39+4	35	4648	114,4	40,62937063	2,78	1715,4215	239,8	6,89	58,46	25,11	618,8	10,3	1135
72	39	0	1	0	1	1	1	0	40,28571429	40+2	32	1566	558,6	2,803437164	2,34	1877,8168	417,3	4,9	163,68	20,49	562,1	10	830
73	37	0	1	0	1	0	1	0	36,57142857	36+4	28,42857143	1054			2,33	1772,7593	377,6	4,67	161,83	21,33	429,2	6,29	619
76	39	1	1	1	1	0	1	0	36	36+0	26,85714286	1526	158,3	9,639924195	3	3424,7016	718,22	4,67	430,93	24,44	417,6	5,79	465
79	37	0	1	0	1	0	0	0	38,71428571	38+5	31,71428571	3188	1059	3,010387158	3,22	2650,6554	365,54	6,89	99,69	26,11	468,4	4,95	1045
82	36	0	0	0	0	0	1	1	38,28571429	38+2	37,14285714	1947	961,7	2,024539877	2	1653,822	309,39	4,83	109,79	21,33	191,4	9,64	990
83	27	0	1	1	1	1	0	1	38,57142857	38+4	34,57142857	1474	1276	1,155172414	2,22	1898,0694	429,53	4,56	188,2	20,22	510,7	5,02	1005
85	43	0	1	0	1	0	1	0	37,42857143	37 + 3	33,14285714	2166	136,2	15,9030837	2,24	1371,2024	271,64	5,25	90,41	20,77	306,6	6,92	1154
89	32	0	1	1	1	0	1	1	37,85714286	37 + 6	32,85714286	1453	650,2	2,234697016	2,56	2618,791	580,77	4,56	290,39	20,33	488,6	5,77	955
90	34	0	1	1	1	1	1	0	38	38+0	38	6394	97,7	65,44524053	3	1979,3962	315,57	6,22	98,31	24,89	420,5	5,62	855
91	29	0	1	0	1	0	0	0	40,42857143	40 + 3	27,28571429	2234	1002	2,229540918	2,67	2641,3137	482,88	5,11	198,35	22,78	584,3	14,24	1840
92	37	0	1	1	1	0	0	1	38,28571429	38 + 2	38,28571429	5196	198,3	26,20272315									1140
94	31	0	1	1	1	1	1	1	39,42857143	39 + 3	28,42857143	1207	622,9	1,937710708	3,22	2388,7993	311,66	7,01	82,48	27,59	487,4	29,19	752
98	32	0	1	0	1	1	0	0	37,28571429	37 + 2	33,14285714	3026	344,2	8,791400349	2,67	1784,9524	315,1	5,78	103,16	22,44	430,5	12,05	1140
102	35	1	1	0	1	0	1	0	35,71428571	35+5	28,57142857	1806	5781	0,312402698	2	2083,2	464,18	4,33	198,93	19,83	565,3	6,63	890
103	28	0	0	0	0	0	1	0	37,85714286	37+6	29,71428571	2161	277,3	7,793003967	2,71	2086,0356	393,33	5,73	133,47	23,13	503,4	10,49	1198
105	30	0	1	1	1	1	1	1	40,28571429	40+2	24,71428571	1310	328,6	3,98660986	2,05	1820,5411	394,84	4,73	147,78	20,42	466,5	4,14	800
111	37	1	1	1	1	1	1	0	38	38+0	38	13591	52,6	258,3840304	2,81	2086,4762	307,32	6,04	95,83	26,3	505,5	14,28	176
114	38	0	1	1	1	0	1	1	39,42857143	39 + 3	39,42857143	3847	267,8	14,36519791	2,67	2264,6632	336,98	6,22	95,07	24,89			
116	40	0	1	0	1	0	1	0	40,57142857	40 + 4	31,71428571	1680	735,2	2,285092492	2,65	2595,2654	466,29	5,32	174,52	23,92	546,5	11,97	740
118	38	0	1	0	1	0	0	0	40	40+0	29,85714286				2,56	1644,4963	294,03	5,67	94,77	22,33	522,6	6,73	630
121	32	0	1	0	1	0	0	0	38,14285714	38 + 1	28,14285714	2138	1268	1,686119874	2,67	1854,2679	261,51	6,78	63,74	24,89	676,6	20,38	725
122	30	0	1	1	1	1	1	1	35,28571429	35 + 2	27	497	246,7		2,02	2431,5023	478,79	4,25	216,26	23,28	676,6	20,38	725
124	36	0	1	1	1	0	0	1	40,42857143	40 + 3	36,14285714	3141	163,6	19,1992665	2,67	2141,2671	345,85	6	103,75	24	757,7	10,2	1183
125	31	0	1	0	1	0	0	0	40,85714286	40 + 6	27,71428571	1686	749,9	2,248299773	2,21	2029,2058	445,12	4,78	177,25	20,73	386,7	8,61	785
127	29	0	1	0	1	0	0	0		RG	28	2771	531,6	5,212565839	3,11	2490,0372	424,19	6	147,09	24,14	600	5,66	695
129	32	0	0	0	0	1	0	0	41,28571429	41+2	33,14285714	1108	383,1	2,892195249	2,56	1946,5724	354,7	5,33	128,14	23,44	597,5	5,51	574
131	42	0	1	0	1	0	0	0		RG	31,71428571	3259	640,4	5,089006871	2,76	1861,9054	279	6,44	75,94	24,81	426,6	10,56	850
133	38	0	1	1	1	0	1	1	38	38 + 0	35,85714286	1058	133,3	7,936984246	2,55	3096,2457	599,55	4,89	255,89	22,18	627,1	9,2	560
134	40	0	0	1	0	0	1	0	39,71428571	39+5	34,28571429	3504	329,8	10,62462098	3,33	2021,708	278,03	7,22	71,63	26,67	529,7	23,48	1036
138	37	0	1	0	1	0	0	0	38,57142857	38 + 4	35,28571429	3309	384,9	8,597038192	2,67	1585,6329	248,4	6,44	65,94	22,44	610,2	35,15	625
139	41	0	1	0	1	0	0	0	37,71428571	37 + 5	27,85714286	950	624,7	1,52072995	2,33	1770,4865	360,35	5	134,93	21,36	521,5	5,84	695
140	30	0	1	1	1	0	0	0	39,14285714	39 + 1	33,14285714	946	796,1	1,188292928	2,05	1777,7924	420,53	4,06	209,86	20,09	369,8	8,69	645
141	28	0	1	0	1	1	0	0	40	40+0	24,85714286	1655	545,6	3,033357771	2,33	1789,3618	326,31	5,44	105,29	22	482,8	5,76	770
149	31	0	1	1	1	0	1	1	38,85714286	38 + 6	26,42857143	1539	223,9	6,873604288	3,11	1976,0995	275,36	6,89	72,97	24,48	407,8	5,48	515
152	41	1	1	1	1	1	0	0	37,42857143	37 + 3	32,85714286	8854	108,2	81,82994455	2,58	2098,321	409,44	5,14	161,34	23,39	429,7	12,79	1303
153	36	0	0	0	0	0	1	0	39,14285714	39 + 1	36,57142857	6810			2,33	1736,0092	317,71	5,33	105,9	21,11	473,5	11,57	1250
154	30	0	1	1	1	0	0	0	37,28571429	37 + 2	24,14285714	483	215,8	2,238183503	2,33	2109,3583	377,14	5,33	125,71	23,11	348,6	9,36	950
157	40	0	1	0	1	0	0	0	38,14285714	38 + 1	28	2318	778,5	2,977520873	3,33	1884,2872	292,74	7,01	79,72	24,37	593,3	5,76	505
161	34	0	0	1	0	1	1	1	39,28571429	39 + 2	34,57142857	1514	290,3	5,215294523	2,67	2740,7194	510,69	5,33	191,51	23,44	316,2	5,93	1450
162	37	0	1	0	1	0	0	0	35,28571429	35 + 2	34	6023	96,2	62,60914761	3,03	2171,2666	320,51	6,37	96,98	25,74	644,8	13,35	1600
163	42	0	1	0	1	0	0	0	36,14285714	36 + 1	27,71428571	1572	642,9	2,445170322	3	2155,0258	423,9	5,67	158,96	23,11	449,8	9,91	945
165	32	0	0	0	0	0	0	1	36,71428571	36 + 5	31	1551	368,1	4,213528932	3,33	2088,5119	362,65	6,22	126,09	23,11	496,6	11,86	510
167	35	0	1	1	1	1	0	1	38,57142857	38 + 4	33,42857143	1433	332,4	4,311070999	2,44	1713,9938	399,96	4,67	180,87	20,22	376	19,15	715
172	40	0	1	1	1	0	1	0	39,14285714	39 + 1	30,14285714	1243	631,8	1,967394745	2,56	2473,2077	501,52	4,89	214,94	23,11	411,9	9,7	530
173	36	0	1	0	1	1	0	0	40,14285714	40 + 1	24,71428571	1620			2,33	1727,5411	337,01	5,56	105,04	21,11	311,9	8,04	1132
174	38	1	1	0	1	0	0	0	39,71428571	39 + 5	33,14285714	1672	362	4,61878453	2,67	2444,7524	494,75	5,22	194,68	21,22	523,1	7,25	966
179	38	0	1	0	1	1	0	0	40,14285714	40+1	37,14285714	3852	385,6	9,989626556	3,33	1903,452	318,59	6,33	106,2	24,11	595,5	11,57	2395
180	30	0	1	0	1	0	0	0	40,85714286	40 + 6	37,28571429	978	1468	0,666212534	2,14	2030,4527	399,61	5,04	139,36	20,84	683,9	25,57	3120
182	37	0	1	1	1	1	0	1	40	40 + 0	28,85714286	785	201,7	3,891918691	3	2135,7414	378,26	5,78	136,7	24,56	493	7,51	880

186	27	0	1	1	1	0	0	0	40,28571429	40 + 2	29	2480	353,7		2,24	1857,4621	379,65	4,91	142,12	20,94	433	7,03	1202
188	34	0	1	0	1	0	0	0	39	39+0	33,42857143	897	959,7	0,934667083	2,89	2270,2738	475,97	5,33	195,76	21,22	516	8,11	1065
190	37	0	1	1	1	0	1	0	38,71428571	38 + 5	34	1539	283,1		2,56	2514,4966	529,27	5	217,93	21,33	583,5	9,67	945
71	26	0	1	0	1	0	1	0	39,57142857	39+4	39,57142857	16069	75,7	212,2721268	2,67	2043,5539	289,77	6,33	79,03	27,22	559,7	10,6	1160
72	39	0	1	0	1	1	1	0	40,28571429	40+2	37,28571429	2326	220,5	10,54875283	2,67	1844,7227	327,26	6,01	97,92	24,39	456,8	7,29	1255
73	37	0	1	0	1	0	1	0	36,57142857	36+4	30,42857143	1441	221	6,520361991	2,33	1722,4491	361,71	5	135,64	21,5	412,6	8,33	747
76	39	1	1	1	1	0	1	0	36	36+0	30,85714286	2098	53,6		2,78	2629,4312	566,72	5	256,3	21,11	516,6	5,62	715
83	27	0	1	1	1	1	0	1	38,57142857	38+4	38,57142857	2017	955,1	2,111820752	2,11	1960,239	441,54	4,22	211,17	20,33	715,7	6,2	1220
85	43	0	1	0	1	0	1	0	37,42857143	37 + 3	37,14285714	3970	86,6	45,84295612	2,24	1548,112	299,37	4,92	112,09	21,27	444,8	9,46	1493
89	32	0	1	1	1	0	1	1	37,85714286	37 + 6	36,85714286	1862	517,5	3,6	2,67	2501,0906	512,91	4,56	274,19	22,33	505,7	6,78	880
91	29	0	1	0	1	0	0	0	40,42857143	40 + 3	31,28571429	2147	1159	1,9	3,22	2635,2933	436,54	5,89	163,7	25,56	620,3	12,29	2040
94	31	0	1	1	1	1	1	1	39,42857143	39 + 3	34,42857143	2397	447,1	5,36121673	3,56	2302,7787	345,19	6,67	111,32	26,89	538,6	5,71	716
102	35	1	1	0	1	0	1	0	35,71428571	35+5	32,57142857	2920	5897	0,495167034	2,22	2150,8396	446,7	4,67	183,24	21	539,1	6,77	865
103	28	0	0	0	0	0	1	0	37,85714286	37+6	33,71428571	4772	157,5	30,2984127	2,55	2067,0852	339,82	5,89	101,53	24,14	359,2	12,22	1722
105	30	0	1	1	1	1	1	1	40,28571429	40+2	33,71428571	1824	215,7	8,456189152	2,5	1829,1052	373,3	5,17	139,72	20,87	439,5	5,7	920
121	32	0	1	0	1	0	0	0	38,14285714	38 + 1	32,14285714	2386	1150	2,074782609	2,67	2006,1775	372,58	5,67	124,19	22,89	617,6	12,36	1095
122	30	0	1	1	1	1	1	1	35,28571429	35 + 2	32,14285714	980	238,8	4,103852596	2,58	1963,3875	397,68	5,03	163,64	22,05	617,6	12,36	1095
124	36	0	1	1	1	0	0	1	40,42857143	40 + 3	40,14285714	7339	113,7	64,54705365	2,67	2445,6267	377,61	6	113,28	24,67	928,7	8,79	1360
125	31	0	1	0	1	0	0	0	40,85714286	40 + 6	31,71428571	2019	729,9	2,766132347	2,22	1722,3854	374,39	4,78	148,27	19,78	524,9	11,91	1100
127	29	0	1	0	1	0	0	0		RG	32	2221	1552	1,431056701	2,89	2250,2068	400,78	5,67	144,83	24	600,2	8,63	1050
129	32	0	0	0	0	1	0	0	41,28571429	41+2	41,14285714	1927	132,8	14,51054217	2,67	2150,9916	369,12	5,44	133,39	24,33	424,7	8,61	1475
131	42	0	1	0	1	0	0	0		RG	35,71428571	4073	425,3	9,576769339	2,99	1860,615	283,65	6,55	79,86	23,92	448,3	10,22	920
138	37	0	1	0	1	0	0	0	38,57142857	38 + 4	38,57142857	4329	276,3	15,66775244	2,56	1912,569	306,33	5,89	91,9	23,67	489,7	11,94	1190
139	41	0	1	0	1	0	0	0	37,71428571	37 + 5	32,85714286	982	942,9	1,041467812	2,67	1458,921	274,54	5,56	95,32	21,44	522	7,47	1205
140	30	0	1	1	1	0	0	0	39,14285714	39 + 1	38,14285714	2363	237,5	9,949473684	3,23	2039,1569	342,26	6,34	110,24	23,93	553,9	15,14	1150
141	28	0	1	0	1	1	0	0	40	40+0	32,85714286	2424	385,2	6,292834891	2,67	2043,751	378,31	5,67	126,1	21,67	729,4	9,7	870
149	31	0	1	1	1	0	1	1	38,85714286	38 + 6	30,42857143	1532	284,6	5,382993675	2,89	1931,7991	286,14	6,56	78,04	24	376,7	4,9	420
152	41	1	1	1	1	1	0	0	37,42857143	37 + 3	34,85714286	12233			2,47	2210,9008	448,66	4,58	212,46	23,05	317	9,94	1415
153	36	0	0	0	0	0	1	0	39,14285714	39 + 1	38,57142857				2,33	1698,079	324,15	5,22	112,57	20,56	889,5	60	1442
154	30	0	1	1	1	0	0	0	37,28571429	37 + 2	27,14285714	493	333,5	1,47826087	2,33	1929,073	355,96	5,33	118,65	22,56	385,1	6,01	935
157	40	0	1	0	1	0	0	0	38,14285714	38 + 1	32	2710	672,6	4,029140648	2,78	1744,9568	309,03	6	96,2	22,56	493,6	6,71	730
161	34	0	0	1	0	1	1	1	39,28571429	39 + 2	37,14285714	1839	225	8,173333333	3	2788,992	475,88	5,67	178,45	24,89	438,3	7,81	1714
162	37	0	1	0	1	0	0	0	35,28571429	35 + 2	35,28571429	9859	62,6	157,4920128	2,36	2066,0829	416,08	4,7	178,01	23,07	1040,3	43,51	6710
165	32	0	0	0	0	0	0	1	36,71428571	36 + 5	35,85714286	3076	155,4	19,79407979	2,78	1816,9157	300,28	6,22	87,46	23,33	451,2	9,35	615
172	40	0	1	1	1	0	1	0	39,14285714	39 + 1	34,14285714	1378	386	3,569948187	2,67	2527,1873	486,2	5,11	199,98	22,67	701,3	16,27	620
173	36	0	1	0	1	1	0	0	40,14285714	40 + 1	28,71428571	1499	337,3	4,444115031	2,56	1551,9607	274,88	6,11	77,64	21,44	429,2	8,57	1624
174	38	1	1	0	1	0	0	0	39,71428571	39 + 5	36,57142857	3620	174	20,8045977	3,17	2711,4492	475,62	6	168,95	24	682,5	9,81	1245
180	30	0	1	0	1	0	0	0	40,85714286	40 + 6	40,85714286	2660	253,4	10,49723757	2,59	1980,7802	427,52	5,15	168,04	20,29	563	11,87	3970
182	37	0	1	1	1	1	0	1	40	40 + 0	40	3600	68,6	52,47813411	3,11	2081,896	325,44	6,33	101,13	25,22	588,7	8,67	1185
186	27	0	1	1	1	0	0	0	40,28571429	40 + 2	34	2391	318,3		2,24	2057,2066	451,18	4,58	193,03	20,44	528,8	6,71	980
188	34	0	1	0	1	0	0	0	39	39+0	36,42857143	1794	875,9	2,048179016	3	2107,2385	413,51	5,78	149,46	21,78	645,7	12,42	1360
73	37	0	1	0	1	0	1	0	36,57142857	36+4	34,42857143	3098	102,4	30,25390625	2,33	1963,1887	355,54	5,5	112,86	23	485,1	7,71	1270
85	43	0	1	0	1	0	1	0	37,42857143	37 + 3	37,28571429	4048	59,8	67,69230769	1,91	1578,7427	316,27	4,58	118,42	20,49	541,5	12,79	2730
91	29	0	1	0	1	0	0	0	40,42857143	40 + 3	35,57142857	4658	214,2	21,7	4	2675,5943	384,72	7,33	115,42	25,67	538,7	10,58	1765
102	35	1	1	0	1	0	1	0	35,71428571	35+5	35,57142857	8178	2102	3,8905804	2,33	2241,6743	468,52	4,44	223,46	21,67	605,4	10,58	1191
105	30	0	1	1	1	1	1	1	40,28571429	40+2	36,14285714	3645	117	31,15384615	2,5	1697,0971	322,45	5,84	96,55	20,98	435	5,9	1080
121	32	0	1	0	1	0	0	0	38,14285714	38 + 1	38,14285714	6097	452,3	13,47999116	4,11	1764,2969	207,42	9	42,49	28,44	688,7	16,02	1635
125	31	0	1	0	1	0	0	0	40,85714286	40 + 6	40,85714286	8472	85,2	99,43661972	1,67	2213,1602	396,84	4,56	137,74	21,67	614,9	11,71	1433
139	41	0	1	0	1	0	0	0	37,71428571	37 + 5	37,71428571	3010	432,9	6,953106953	3	939,7048	149,92	7	37,48	22,56	369,5	10,26	1845
141	28	0	1	0	1	1	0	0	40	40+0	38,85714286	6535	134,6	48,551263	2,67	2119,8804	378,89	5,67	126,3	22,11	778,4	10,76	960
149	31	0	1	1	1	0	1	1	38,85714286	38 + 6	34,42857143	2251	104,3	21,58197507	3,33	1918,8087	275,43	7	75,12	24,67	519,4	46,59	1005
152	41	1	1	1	1	1	0	0	37,42857143	37 + 3	37,42857143	18410	59,5	309,4117647	2,58	2021,6534	361,23	4,92	154,57	24,78	477,5	14,79	4686

154	30	0	1	1	1	0	0	0	37,28571429	37 + 2	31,14285714	661	370,4	1,784557235	2,67	2104,7226	336,2	6	101,95	24,78	445,1	7,59	980
157	40	0	1	0	1	0	0	0	38,14285714	38 + 1	34	3338	584,8	5,707934337	3	1697,4666	276,96	6,44	80,66	23,56	650	9,1	700
161	34	0	0	1	0	1	1	1	39,28571429	39 + 2	39,28571429	3756	74,5	50,41610738	2,33	2894,8725	617,83	4,33	308,91	22,44	751,6	18,56	2305
172	40	0	1	1	1	0	1	0	39,14285714	39 + 1	38,14285714	3142	299,7	10,48381715	3	2544,7169	436,03	5,67	163,51	23,89	656,4	10,51	785
173	36	0	1	0	1	1	0	0	40,14285714	40 + 1	32,42857143	1580	343,4	4,60104834	2,44	1645,1889	291,94	5,89	84,87	21,33	711,6	36,16	1613
186	27	0	1	1	1	0	0	0	40,28571429	40 + 2	38	3079	249,1		2,24	1965,4662	364,7	5,25	121,36	21,94	642,8	8,45	1180
73	37	0	1	0	1	0	1	0	36,57142857	36+4	36,57142857	4845	84,5	57,33727811	2,33	2053,5192	453,77	4,67	194,47	20,44	739,1	15,08	2545
91	29	0	1	0	1	0	0	0	40,42857143	40 + 3	38,71428571	4030	349,7	11,5	3,67	2629,3697	337,39	7	101,22	30,44	424,2	12,11	1365
102	35	1	1	0	1	0	1	0	35,71428571	35+5	35,71428571	7251	1881	3,854864434	2	2072,025	421,52	4,33	180,65	22	536,6	13,07	1020
105	30	0	1	1	1	1	1	1	40,28571429	40+2	40,14285714	4057	85,7	47,33955659	2,28	1922,1267	379,88	5,06	137,01	20,98	437	9,4	1300
141	28	0	1	0	1	1	0	0	40	40+0	40	5847	169	34,59763314							1294,9	55,13	1105
149	31	0	1	1	1	0	1	1	38,85714286	38 + 6	38,71428571	5665	63,8	88,79310345	3	2154,4697	259,9	7,22	61,7	26,67	299,9	5,62	1015
154	30	0	1	1	1	0	0	0	37,28571429	37 + 2	35,14285714	1113	263,8	4,219105383	2,56	1788,8822	331,67	5,33	119,79	22,33	575,2	6,88	1200
172	40	0	1	1	1	0	1	0	39,14285714	39 + 1	38,71428571				2,67	2531,5597	455,99	5,11	187,77	24,89	697,7	13,28	1083
173	36	0	1	0	1	1	0	0	40,14285714	40 + 1	38,71428571	3724	148,5	25,07744108	2,56	1907,1997	300,65	6,44	78,41	22,78	684,5	14,1	1926
186	27	0	1	1	1	0	0	0	40,28571429	40 + 2	38,71428571	3903	161,2		2,24	1852,4297	392,37	4,8	154,16	20,72	341,9	14,17	1312
91	29	0	1	0	1	0	0	0	40,42857143	40 + 3	40,42857143	7700	97,7	79,3	3,56	2685,1081	383,65	6,67	123,64	28	589	13,44	1565
173	36	0	1	0	1	1	0	0	40,14285714	40 + 1	40,14285714	5843	80,3	72,76463263	2,78	1930,0767	327,42	6,44	90,58	22,44	710,2	15,22	2511
72	39	0	1	0	1	1	1	0	40,28571429	40+2	40,71428571	471	9,7	48,55670103	1,67	1706,7895	440,48	4,01	188,29	16,6	586,7	9,51	560
76	39	1	1	1	1	0	1	0	36	36+0	36	4023	51,2	78,57421875	2,33	2394,8764	518,68	4,33	259,34	22,11	719,2	7,75	2755
80	28	0	0	1	0	0	0	0	40,57142857	40+4	40,85714286	6533	39,8	164,1457286	3,33	1765,2202	236,78	7,11	62,86	27,33	709,4	22,1	
90	34	0	1	1	1	1	1	0	38	38+0	38,42857143	550	13	42,3	3	1674,5083	320,73	5,56	126,3	21,44	521,6	4,85	1355
104	26	0	1	1	1	0	1	1	40,14285714	40+1	40,57142857	268	16,6	16,14457831	3	2867,1688	485,35	5,83	172,3	21	567,2	5,52	1330
106	30	0	1	0	1	1	0	0	41	41+0	41,14285714	1098	25,2	43,57142857	2,56	1919,5116	380,22	4,78	172,44	24,11	700,1	9,89	
107	37	0	0	0	0	1	0	0	32,28571429	32 + 2	32,71428571	209	15,5	13,48387097	2,33	1759,5103	386,65	4,78	158,9	20,33	717	5,24	1180
111	37	1	1	1	1	1	1	0	38	38+0	38,57142857	509	17,7	28,75706215	3,92	1836,179	237,61	7,93	59,3	26,13	718,5	5,07	2435
121	32	0	1	0	1	0	0	0	38,14285714	38 + 1	38,42857143	882	21,4	41,21495327	3,67	1611,5783	204,56	7,67	51,14	27,33	618	5,51	960
130	25	0	0	1	0	1	0	0	37,28571429	37 + 2	37,71428571	332	10,7	31,02803738	2,33	2225,8948	452,78	4,67	194,05	21,22	360,3	5,36	850
135	35	0	0	0	0	0	0	0	36,42857143	36 + 3	36,85714286	347	18,1	19,17127072	3	1741,4906	327,29	6	109,1	21,44	646	5,74	1160
140	30	0	1	1	1	0	0	0	39,14285714	39 + 1	39,28571429	670	24,8	27,01612903	2,34	2119,3825	455,28	4,34	227,28	22,03	474,5	5,64	1740
144	42	0	0	0	0	0	0	0	40,28571429	40 + 2	40,42857143	646	16	40,375	2,56	1988,9581	462,49	4,67	220,25	20,11	482,1	5,2	1345
145	34	0	1	0	0	0	0	0	38	38 + 0	38,28571429	1123	20,1		2	2690,7576	532,26	4	266,13	23,11	350,4	5,19	1880
156	36	0	0	0	0	0	0	1	41,14285714	41 + 1	41,42857143	794	16,5	48,12121212	2,78	2459,713	473,77	5	214,51	23,33	645,2	8,65	8995
157	40	0	1	0	1	0	0	0	38,14285714	38 + 1	38,71428571	3733	384,8	9,701143451	3,11	1636,4297	242,23	6,78	66,06	24,44	493,1	11,38	1432
162	37	0	1	0	1	0	0	0	35,28571429	35 + 2	36,14285714	492	8	61,5	2,67	2022,7671	408,75	5,22	160,54	22,11	383,2	7,87	1135
170	40	0	1	0	1	0	0	0	41,14285714	41 + 1	41,42857143	631	16,5	38,24242424	4,67	1069,143	102,69	9,56	21,02	31,22	530,7	11,03	1160
182	37	0	1	1	1	1	0	1	40	40 + 0	40,14285714	768	21,6	35,55555556	2,67	2333,3567	493,08	4,67	246,54	22,67	674,3	5,05	1195
184	31	0	0	0	0	0	1	0	40,42857143	40 + 3	40,71428571	580	6,6	87,87878788	2	1638,8495	358,92	4	179,46	21,11	897,8	5,14	975
